# GDF15 promotes weight loss by enhancing energy expenditure in muscle

**DOI:** 10.1038/s41586-023-06249-4

**Published:** 2023-06-28

**Authors:** Dongdong Wang, Logan K. Townsend, Geneviève J. DesOrmeaux, Sara M. Frangos, Battsetseg Batchuluun, Lauralyne Dumont, Rune Ehrenreich Kuhre, Elham Ahmadi, Sumei Hu, Irena A. Rebalka, Jaya Gautam, Maria Joy Therese Jabile, Chantal A. Pileggi, Sonia Rehal, Eric M. Desjardins, Evangelia E. Tsakiridis, James S. V. Lally, Emma Sara Juracic, A. Russell Tupling, Hertzel C. Gerstein, Guillaume Paré, Theodoros Tsakiridis, Mary-Ellen Harper, Thomas J. Hawke, John R. Speakman, Denis P. Blondin, Graham P. Holloway, Sebastian Beck Jørgensen, Gregory R. Steinberg

**Affiliations:** 1 Centre for Metabolism, Obesity and Diabetes Research, McMaster University, Hamilton, Ontario Canada; 2 Division of Endocrinology and Metabolism, Department of Medicine, McMaster University, Hamilton, Ontario Canada; 3 Department of Human Health and Nutritional Sciences, University of Guelph, Guelph, Ontario Canada; 4 Department of Pharmacology-Physiology, Centre de Recherche du Centre Hospitalier Universitaire de Sherbrooke, Université de Sherbrooke, Sherbrooke, Quebec Canada; 5 Global Obesity and Liver Disease Research, Global Drug Discovery, Novo Nordisk, Maaloev, Denmark; 6 Department of Biomedical Sciences, Faculty of Health and Medical Sciences, University of Copenhagen, Copenhagen, Denmark; 7 Key Laboratory of Geriatric Nutrition and Health, Ministry of Education, Beijing Technology and Business University, Beijing, China; 8 Shenzhen Key Laboratory of Metabolic Health, Center for Energy Metabolism and Reproduction, Shenzhen Institutes of Advanced Technology, Chinese Academy of Sciences, Shenzhen, China; 9 Department of Pathology and Molecular Medicine, McMaster University, Hamilton, Ontario Canada; 10 Department of Biochemistry, Microbiology and Immunology, Faculty of Medicine, University of Ottawa, Ottawa, Ontario Canada; 11 Ottawa Institute of Systems Biology, University of Ottawa, Ottawa, Ontario Canada; 12 Department of Kinesiology and Health Sciences, University of Waterloo, Waterloo, Ontario Canada; 13 Population Health Research Institute, Hamilton Health Sciences and McMaster University, Hamilton, Ontario Canada; 14 Thrombosis and Atherosclerosis Research Institute, McMaster University, Hamilton Health Sciences, Hamilton, Ontario Canada; 15 Department of Health Research Methods, Evidence, and Impact, McMaster University, Hamilton, Ontario Canada; 16 Department of Oncology, McMaster University, Hamilton, Ontario Canada; 17 State Key Laboratory of Molecular Developmental Biology, Institute of Genetics and Developmental Biology, Chinese Academy of Sciences, Beijing, China; 18 School of Biological Sciences, University of Aberdeen, Aberdeen, UK; 19 CAS Center for Excellence in Animal Evolution and Genetics (CCEAEG), Kunming, China; 20 Division of Neurology, Department of Medicine, Centre de Recherche du Centre Hospitalier Universitaire de Sherbrooke, Université de Sherbrooke, Sherbrooke, Quebec Canada; 21 Bio Innovation Hub Transformational Research Unit, Novo Nordisk, Boston, MA USA; 22 Department of Biochemistry and Biomedical Sciences, McMaster University, Hamilton, Ontario Canada

**Keywords:** Obesity, Diabetes, Fat metabolism, Recombinant protein therapy

## Abstract

Caloric restriction that promotes weight loss is an effective strategy for treating non-alcoholic fatty liver disease and improving insulin sensitivity in people with type 2 diabetes^1^. Despite its effectiveness, in most individuals, weight loss is usually not maintained partly due to physiological adaptations that suppress energy expenditure, a process known as adaptive thermogenesis, the mechanistic underpinnings of which are unclear^2,3^. Treatment of rodents fed a high-fat diet with recombinant growth differentiating factor 15 (GDF15) reduces obesity and improves glycaemic control through glial-cell-derived neurotrophic factor family receptor α-like (GFRAL)-dependent suppression of food intake^4–7^. Here we find that, in addition to suppressing appetite, GDF15 counteracts compensatory reductions in energy expenditure, eliciting greater weight loss and reductions in non-alcoholic fatty liver disease (NAFLD) compared to caloric restriction alone. This effect of GDF15 to maintain energy expenditure during calorie restriction requires a GFRAL–β-adrenergic-dependent signalling axis that increases fatty acid oxidation and calcium futile cycling in the skeletal muscle of mice. These data indicate that therapeutic targeting of the GDF15–GFRAL pathway may be useful for maintaining energy expenditure in skeletal muscle during caloric restriction.

## Abstract (web summary)

GDF15 treatment in mice counteracts compensatory reductions in energy expenditure, resulting in greater weight loss and reductions in non-alcoholic fatty liver disease compared to caloric restriction alone.

## Main

GDF15 is highly expressed in the liver and kidneys and is induced in all cell types in response to mitochondrial toxins and endoplasmic reticulum stress (reviewed previously^8–12^). GDF15 was first identified as a soluble factor secreted from macrophages^13^ and cancer cells^14^ and was later shown to induce cachexia^15,16^ and protect mice from obesity and insulin resistance^17,18^. In rodents fed a high-fat diet, treatment with recombinant GDF15 elicits weight loss, reduces liver steatosis and improves glycaemic control^8–12^. These weight-loss effects have been shown to require the hindbrain^19^ and, more specifically, the GDF15 receptor GFRAL^4–7^. In short-term experiments spanning 7–10 days, pair feeding (caloric matching) of vehicle-treated mice showed that weight loss elicited by GDF15 is due to reductions in food intake^4–7^. Importantly, germline *Gdf15*-null mice^20^, liver-targeted *Gdf15-*null mice^21^ and germline *Gfral-*null mice^4–7^ all have modest increases in food intake and adiposity when fed a high-fat diet supporting a physiological role of this pathway in regulating energy balance. These studies have led to the concept that GDF15 signalling through GFRAL reduces body mass and improves glycaemic control primarily through suppression of appetite while having minimal effects on energy expenditure^4–7^.

Obesity results from a caloric imbalance between energy intake and expenditure. Although it is well-established that GDF15 suppresses energy intake in rodents and non-human primates^4–7^, three important distinctions need to be considered before concluding that this is the only mechanism contributing to weight loss. The first and most important is that energy intake, energy expenditure and body weight are interdependent variables that are dynamically linked to each other, in that, reductions in energy intake and weight loss can both lead to reduced energy expenditure^22^. Second, studies with recombinant GDF15 in *Gfral*-null mice were conducted over a relatively short period (7–10 days)^4–7^, which may have been insufficient to detect counter regulatory responses related to the reduction of energy expenditure (that is, adaptive thermogenesis) that typically occur in rodents after longer periods of caloric restriction^23^. Finally, it is now recognized that conducting energy balance experiments in mice housed at room temperature (21 °C), which is below the thermoneutral zone for rodents, stimulates sympathetic drive^24^. This may suppress weight loss elicited by agents that induce futile cycling or stimulate energy expenditure through the β-adrenergic signalling pathway^25^. Collectively, these studies indicate that it is important to consider the interrelationships between caloric intake, duration of intervention and housing temperature when studying weight loss and pharmacological interventions in mice.

## GDF15 reduces obesity more than food restriction

**Fig. 1:**
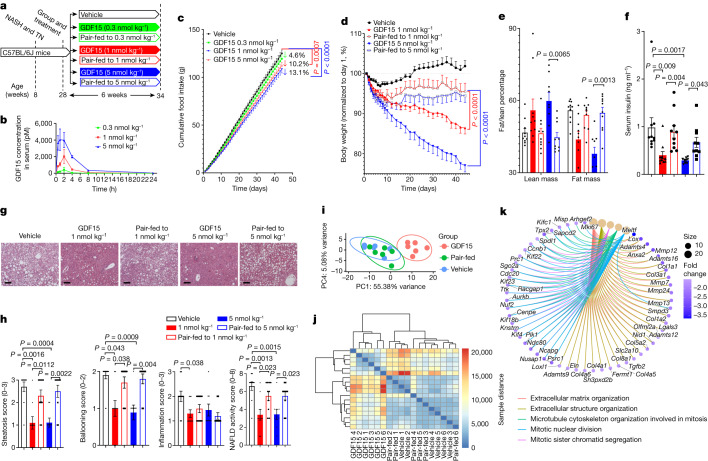
GDF15 reduces obesity, insulin resistance and NASH independently of reductions in food intake. **a**, Experimental schematic. TN, thermoneutrality. **b**, Plasma GDF15 after injection with 0.3, 1 and 5 nmol per kg GDF15. Data are mean ± s.e.m. *n* = 3 mice per group. **c**, Food intake over time. Data are mean ± s.e.m. *n* = 10 mice per group, except for GDF15 (5 nmol per kg), for which *n* = 9 mice. *P* values were calculated using two-way analysis of variance (ANOVA) with Tukey’s multiple-comparison test. **d**, The percentage change in body mass over time. Data are mean ± s.e.m. *n* = 10 mice per group, except for GDF15 (5 nmol per kg), for which *n* = 9 mice. *P* values were calculated using two-way ANOVA with Tukey’s multiple-comparison test. **e**, The percentage of fat/lean mass relative to body mass. Data are mean ± s.e.m. *n* = 10 mice per group, except for GDF15 (5 nmol per kg), for which *n* = 9 mice. *P* values were calculated using two-way ANOVA with Tukey’s multiple-comparison test. **f**, Serum insulin. Data are mean ± s.e.m. *n* = 10 mice per group, except for GDF15 (5 nmol per kg), for which *n* = 9 mice. *P* values were calculated using one-way ANOVA with Tukey’s multiple-comparison test. **g**, Representative images of paraffin-embedded liver sections stained with haematoxylin and eosin (H&E). **h**, From left to right, steatosis score, ballooning score, inflammation score and NAFLD activity score. Data are mean ± s.e.m. *n* = 10 mice per group, except for GDF15 (5 nmol per kg), for which *n* = 9 mice. *P* values were calculated using two-sided unpaired Mann–Whitney *U*-tests. **i**, PCA of liver samples from vehicle-treated and GDF15-treated (5 nmol per kg) mice and pair-fed controls using VST data from DESeq2. *n* = 6 mice per group. **j**, Heatmap of the sample-to-sample distances on the basis of VST data from DESeq2. *n* = 6 mice per group. **k**, Gene-concept network diagram, indicating the corresponding enriched GO terms according to differentially expressed genes (DEGs) between vehicle and GDF15 groups. Source data

**Extended Data Fig. 1:**
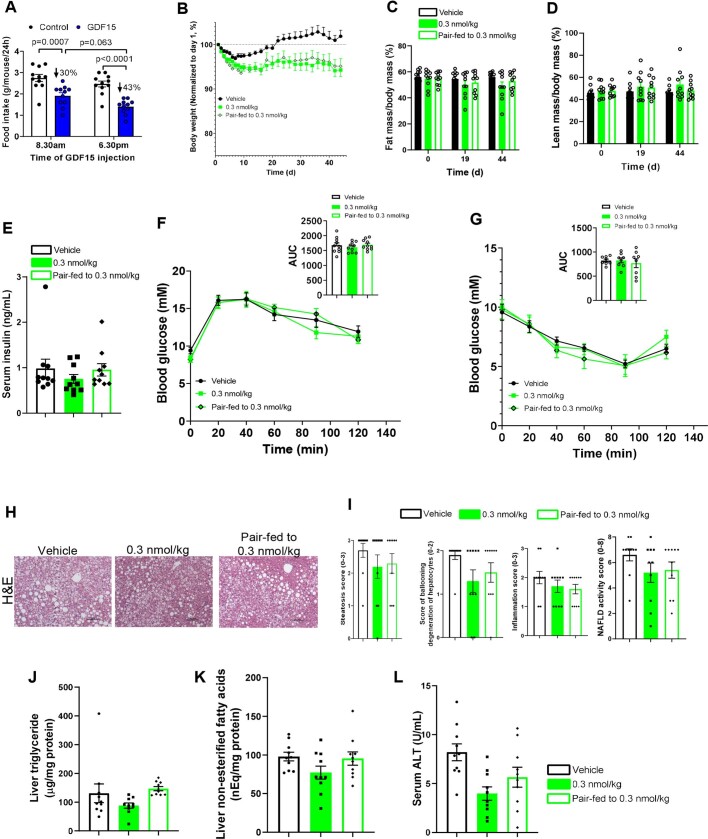
GDF15 at 0.3 nmol/kg has no effects on body weight, body composition, glucose homeostasis and NAFLD. **A**, the influence of injection of mice on food intake at the start of the light/dark-cycle with GDF15 (5 nmol/kg). Data are means ± SEM, n = 10 mice/group. P values by two-way ANOVA with Tukey’s multiple comparisons test. **B**, Body weight. Data are means ± SEM, n = 10 mice/group. **C**, Fat mass/body mass (%). Data are means ± SEM, n = 10 mice/group. **D**, Lean mass/body mass (%). Data are means ± SEM, n = 10 mice/group. **E**, Serum insulin levels. Data are means ± SEM, n = 10 per group. **F**, Glucose tolerance test (GTT). Data are means ± SEM, n = 10 mice/group. **G**, Insulin tolerance test (ITT). Data are means ± SEM, n = 8 mice/group. **H**, Representative images of paraffin-embedded liver sections stained with H&E. **I**, Steatosis score, score of ballooning degeneration of hepatocytes, inflammation score, and NAFLD activity score. Data are means ± SEM, n = 10 mice/group. **J**, liver triglycerides (TG). Data are means ± SEM, n = 10 mice/group. **K**, Liver non-esterified free-fatty acids (NEFA). Data are means ± SEM, n = 10 mice/group. **L**, Serum alanine aminotransferase (ALT). Data are means ± SEM, n = 10 mice/group. Source data

To better understand the mechanisms by which GDF15 may promote weight loss, we studied mice housed at thermoneutrality (29 °C) that were fed a western style diet high in both fat and fructose that promotes obesity, insulin resistance and non-alcoholic steatohepatitis (NASH) with a similar pathological, histological and transcriptional profile to that of human disease development^26^ (Fig. 1a). As liver steatosis in mice is acutely sensitive to changes in caloric intake, we hypothesized that, given the short half-life (2 h in mice) of native human GDF15, treatment at the start of the light-cycle (the time period when mice eat fewer calories) would have a smaller effect on food consumption compared with our previous studies when mice were injected at the start of the dark cycle^4^. Consistent with this hypothesis, compared with the vehicle-treated control, injection of mice at the start of the light cycle with GDF15 (5 nmol per kg) led to a 30% reduction in daily food intake compared with a 43% reduction at the start of the dark cycle (a difference of around 40%; Extended Data Fig. 1a). We subsequently injected mice once daily at the start of the light cycle with either vehicle or recombinant GDF15 at three different doses (0.3, 1 and 5 nmol per kg) for 6 weeks. Individual food intake was measured daily and matched to pair-fed controls. The injection of GDF15 rapidly and dose-dependently elevated serum levels of GDF15 before declining back to the baseline by the start of the dark cycle (Fig. 1b). As expected, chronic daily treatment with GDF15 led to dose-dependent decreases in food intake (Fig. 1c), consistent with previous observations using the same recombinant protein preparation^4^.

**Extended Data Fig. 2:**
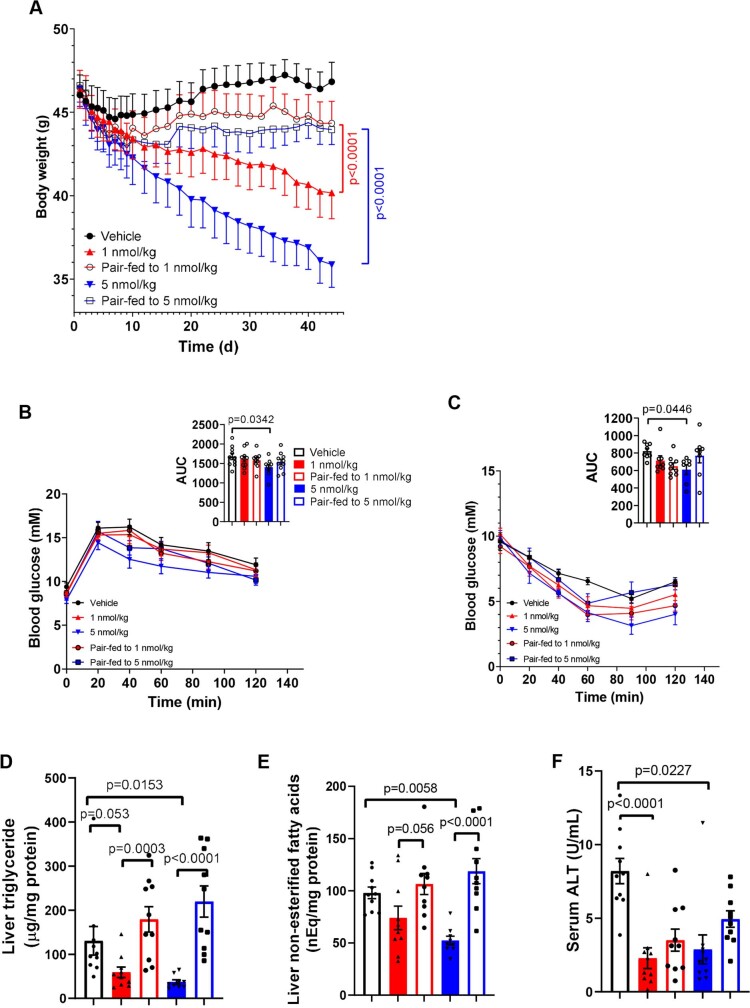
The effect of GDF15 at 1 and 5 nmol/kg on glucose homeostasis, liver TG, NEFA and ALT. **A**, Body weight. Data are means ± SEM, n = 10 mice/group except GDF15 (5 nmol/kg), n = 9 mice. P values by two-way ANOVA with Tukey’s multiple comparisons test. **B**, Glucose tolerance test (GTT). Data are means ± SEM, n = 10 mice/group except GDF15 (5 nmol/kg), n = 9 mice. P values by one-way ANOVA with Dunnett’s multiple comparisons test. **C**, Insulin tolerance test (ITT). Data are means ± SEM, n = 10 mice/group except GDF15 (5 nmol/kg), n = 9 mice. P values by one-way ANOVA with Dunnett’s multiple comparisons test. **D**, Liver triglycerides (TG). Data are means ± SEM, n = 8 mice/group. P values by one-way ANOVA with Dunnett’s multiple comparisons test. **E**, Liver non-esterified free-fatty acids (NEFA). Data are means ± SEM, n = 10 mice/group except GDF15 (5 nmol/kg), n = 9 mice. P values by one-way ANOVA with Dunnett’s multiple comparisons test. **F**, Serum alanine aminotransferase (ALT). Data are means ± SEM, n = 10 mice/group except GDF15 (5 nmol/kg), n = 9 mice. P values by one-way ANOVA with Dunnett’s multiple comparisons test. Source data

GDF15 delivered at 0.3 nmol per kg did not significantly reduce body mass, fat mass, serum insulin, glucose tolerance, insulin resistance, liver histology, liver triglycerides or other plasma variables compared with vehicle-treated or pair-fed controls (Extended Data Fig. 1b–l). When GDF15 was delivered at 1 and 5 nmol per kg, over the first 10 days of treatments, the trajectory of weight loss was similar between GDF15 treatment and pair-fed controls, mirroring previous experiments over this time period^4–7^ (Fig. 1d and Extended Data Fig. 2a). However, after 10 days, the body mass of pair-fed controls did not decrease further, whereas GDF15-treated mice continued to lose weight (Fig. 1d and Extended Data Fig. 2a). By the end of the experiment, the mice that were treated with GDF15 at 1 and 5 nmol per kg had lost 13.6% and 23.0% of their body mass, respectively, compared with around 5% for the pair-fed control mice (Fig. 1d). Importantly, this weight loss was attributed to a reduction in fat mass but not lean mass (Fig. 1e), which is known to be important for maintaining energy expenditure^27^. Consistent with reductions in body mass and adiposity, GDF15 at 1 and 5 nmol per kg lowered serum insulin (Fig. 1f), whereas GDF15 at 5 nmol per kg improved glucose tolerance and insulin sensitivity compared with the vehicle-treated controls (Extended Data Fig. 2b,c). These data indicate that GDF15 in a chronic setting promotes reductions in body mass and reduces insulin resistance to a greater degree than caloric restriction alone.

## GDF15 reduces NASH independently of caloric intake

**Extended Data Fig. 3:**
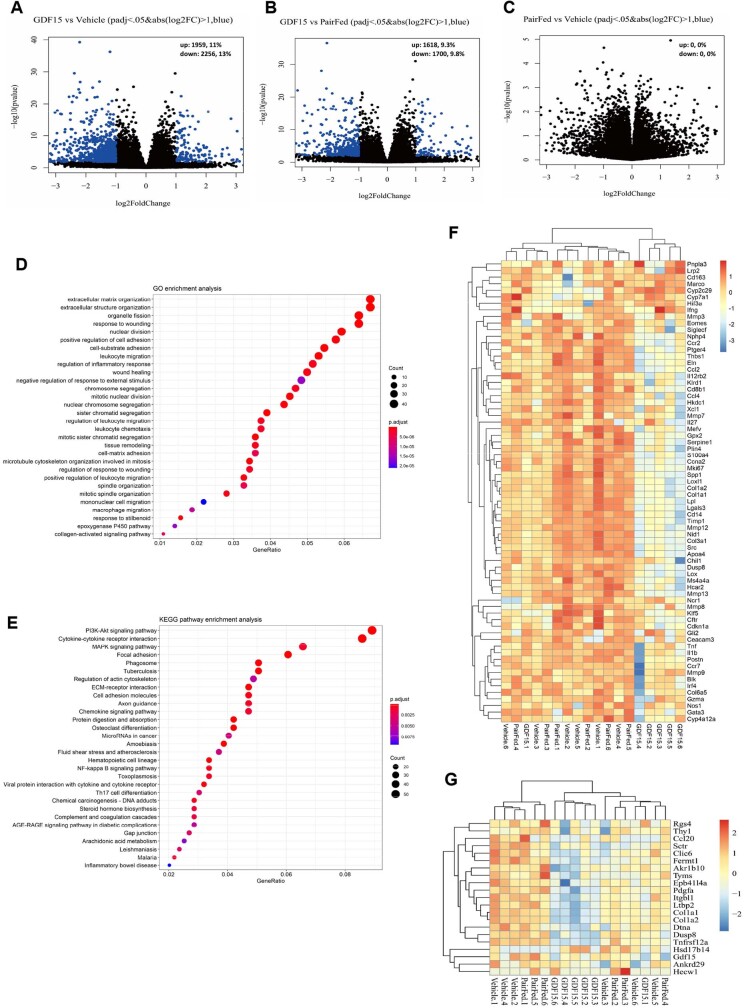
Liver transcriptomic analysis based on RNA-seq data. **A**-**C**, Volcano plot showing differential expression genes (DEGs) identified between GDF15 and vehicle groups (A), GDF15 and pair-fed groups (B), and pair-fed and vehicle groups (C). n = 6 mice/group. Differential expression genes analysis with DEGseq2 is based on Wald test (two-sided, no adjustment). **D**, Top 30 terms in GO annotation involved in DEGs between vehicle and GDF15, n = 6 mice/group. Adjust-p value calculated by Benjamini-Hochberg method. **E**, Top 30 pathway by using KEGG pathway enrichment analysis. Adjust-p value calculated by Benjamini-Hochberg method. **F**, Hierarchical clustering by using the mouse fibrosis panel from NanoString. **G**, A hierarchical clustering of the liver RNA-seq data by using the 25-gene signature established^31^ in humans.

NAFLD is an important factor contributing to insulin resistance^1^. GDF15 (1 and 5 nmol per kg), but not pair-feeding, reduced liver steatosis, ballooning and NAFLD activity scores (Fig. 1g,h). Consistent with these histological changes, GDF15 but not pair-feeding, reduced liver triglycerides, liver non-esterified free-fatty acids and serum alanine aminotransferase (ALT) (Extended Data Fig. 2d–f). Principal component analysis (PCA) using variance stabilizing transformation (VST) data and heat maps of the same-to-sample differences from liver RNA-sequencing (RNA-seq) data showed distinct separation between GDF15-treated (1 nmol per kg) and vehicle-treated and pair-fed controls (Fig. 1i,j). Compared with the vehicle-treated and pair-fed controls, GDF15 elicited many differentially expressed genes (Extended Data Fig. 3a,b) associated with extracellular matrix organization, extracellular structure organization (Fig. 1k and Extended Data Fig. 3d), leukocyte/macrophage migration and phosphatidylinositol 3-kinase–AKT signalling (Extended Data Fig. 3d,e), whereas there were no differences between the vehicle-treated and pair-fed group (Extended Data Fig. 3c). Hierarchical clustering showed that GDF15 changed liver-fibrosis-related transcriptomic signatures compared with the vehicle-treated or pair-fed group (Extended Data Fig. 3f). Furthermore, an established 25-gene signature used to predict NASH progression in humans^28^ found that GDF15 downregulated 20 out of the 25 genes (*Akr1b10*, *Ankrd29*, *Ccl20*, *Clic6*, *Col1a1*, *Col1a2*, *Dtna*, *Dusp8*, *Epb41l4a*, *Fermt1*, *Gdf15*, *Hecw1*, *Itgbl1*, *Ltbp2*, *Pdgfa*, *Rgs4*, *Sctr*, *Thy1*, *Tnfrsf12a* and *Tyms*), while upregulating only 1 gene (*Hsd17b14)* (Extended Data Fig. 3g). Thus, GDF15 reduces NASH independently of reductions in caloric intake.

## GDF15 blocks adaptive thermogenesis with weight loss

**Fig. 2:**
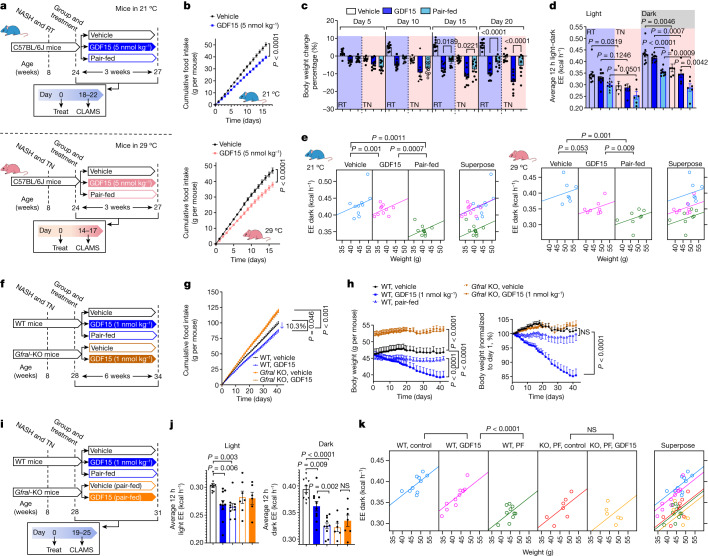
GDF15 increases energy expenditure and reduces body mass through GFRAL. **a**, Experimental schematic. Mice were housed at room temperature (RT; 21 °C) or thermoneutrality (TN, 29 °C). CLAMS,Comprehensive Laboratory Animal Monitoring System. **b**, Cumulative food intake. Data are mean ± s.e.m. *n* = 10 mice per group at room temperature. *n* = 7 mice per group at thermoneutrality. *P* values were calculated using two-way ANOVA with Tukey’s multiple-comparison test. **c**, Percentage body weight change. Data are mean ± s.e.m. *n* = 10 mice per group. *P* values were calculated using one-way ANOVA with Tukey’s multiple-comparison test. **d**, Average energy expenditure. Data are mean ± s.e.m. *n* = 10 mice per group at room temperature. *n* = 6 mice per group at thermoneutrality. *P* values were calculated using one-way ANOVA with Šidák’s multiple-comparison test. **e**, ANCOVA using body mass as a covariate (two-sided without adjustment). *n* = 10 mice per group at room temperature and *n* = 6 mice per group at thermoneutrality. **f**, Experimental schematic for the effect of GDF15 on WT and *Gfral*-KO mice. EE, energy expenditure. **g**, Cumulative food intake. Data are mean ± s.e.m. *n* = 10 mice per group. *P* values were calculated using two-way ANOVA with Tukey’s multiple-comparison test. **h**, Body weight and percentage change over time. Data are mean ± s.e.m. *n* = 10 mice per group. *P* values were calculated using two-way ANOVA with Tukey’s multiple-comparison test. **i**, Experimental schematic for the effects of GDF15 and matched caloric restriction on energy expenditure in WT and *Gfral*-KO mice. **j**, The average energy expenditure during a 12 h–12 h light–dark cycle. Data are mean ± s.e.m. *n* = 10 (WT, vehicle; WT, GDF15; and WT pair-fed), *n* = 7 (KO, pair-fed) and *n* = 6 (KO, GDF15) mice. *P* values were calculated using one-way ANOVA with Šidák’s multiple-comparison test. NS, not significant. **k**, ANCOVA using body mass as a covariate and treatment as a fixed factor (two-sided without adjustment). *n* = 10 (WT, vehicle; WT, GDF15; and WT, pair-fed (PF)), *n* = 7 (KO, pair-fed) and *n* = 6 (KO, GDF15) mice. Images of mice generated using BioRender.com. Source data

To examine whether the effects of GDF15 treatment compared with pair-fed controls was dependent on housing temperature, we subsequently completed matched experiments in mice housed at 21 °C and 29 °C, respectively (Fig. 2a). Over 16 days, GDF15 treatment led to a similar reduction in cumulative food intake at 21 and 29 °C (21.3% versus 19.2%) (Fig. 2b). GDF15-induced weight loss was not different from pair-fed controls after 5 or 10 days at 21 or 29 °C (Fig. 2c). After 10 days, regardless of housing temperature, GDF15-treated mice continued to lose weight, whereas pair-fed control mice did not (Fig. 2c).

To study how GDF15 promoted weight loss, mice were placed into metabolic cages. There was no change in physical activity between the groups (Supplementary Fig. 1). However, increased weight loss with GDF15 treatment was associated with the maintenance of energy expenditure during the dark cycle compared with pair-fed control mice, whose energy expenditure was reduced even after correcting for body mass by analysis of covariance (ANCOVA; Fig. 2d,e). This effect of GDF15 to maintain energy expenditure was evident regardless of housing temperature. As anticipated, energy expenditure was significantly higher at 21 °C compared with at 29 °C for all conditions (Fig. 2d). These data indicate that, in mice housed at 21 °C or 29 °C, treatment with GDF15 for more than 10 days promotes weight loss compared with caloric restriction by maintaining energy expenditure.

**Extended Data Fig. 4:**
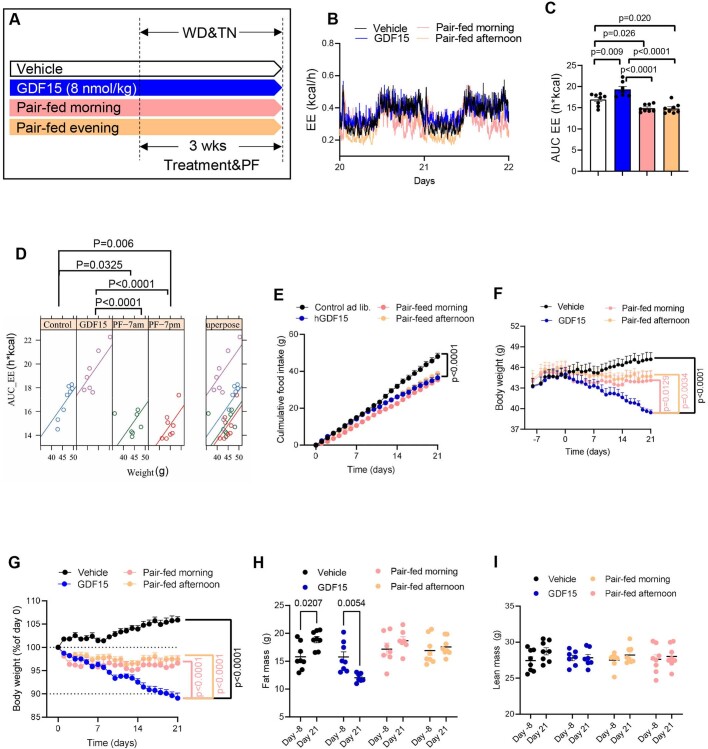
GDF15 increases energy expenditure independently on the time of feeding. **A**, Experimental scheme for the effects of GDF15 and matched caloric restriction (the “pair-fed morning” group: fed at start of light cycle (0600-0700h); the “pair-fed evening” group: fed at start of dark cycle (1800–1900h)) on body mass and energy expenditure (EE) in mice. **B**, Average energy expenditure curves. **C**, AUC of average energy expenditure. Data are means ± SEM, n = 8 mice/group except GDF15 group, n = 7 mice. P values by one-way ANOVA with Tukey’s multiple comparisons test. **D**, ANCOVA of total energy expenditure against body weight of mice using body mass as a covariate and treatment as a fixed factor. n = 8 mice/group except GDF15 group, n = 7 mice. **E**, Cumulative food intake. Data are means ± SEM, n = 8 mice/group. P values by two-way ANOVA with Tukey’s multiple comparisons test. **F**, Body weight. Data are means ± SEM, n = 8 mice/group except GDF15 group, n = 7 mice. P values by two-way ANOVA with Tukey’s multiple comparisons test. **G**, Body weight percentage (Normalized to day 0). Data are means ± SEM, n = 8 mice/group except GDF15 group, n = 7 mice. P values by two-way ANOVA with Tukey’s multiple comparisons test. **H**, Fat mass. Data are means ± SEM, n = 8 mice/group. P values by one-way ANOVA with Tukey’s multiple comparisons test. **I**, Lean mass. Data are means ± SEM, n = 8 mice/group except GDF15 group, n = 7 mice. P values by one-way ANOVA with Tukey’s multiple comparisons test. Source data

Circadian rhythms and time of feeding influence energy expenditure; we therefore examined the effects of GDF15 in relation to two different pair-fed groups (pair-fed morning group, fed at start of light cycle (06:00–07:00); pair-fed evening group, fed at start of dark cycle (18:00–19:00)) performed at a different site (Novo Nordisk, Denmark). The use of this revised protocol at a different location with a higher dose of GDF15 (8 versus 1 nmol per kg) led to increases in energy expenditure while stimulating body mass loss compared with both pair-fed and control groups (Extended Data Fig. 4).

**Extended Data Fig. 5:**
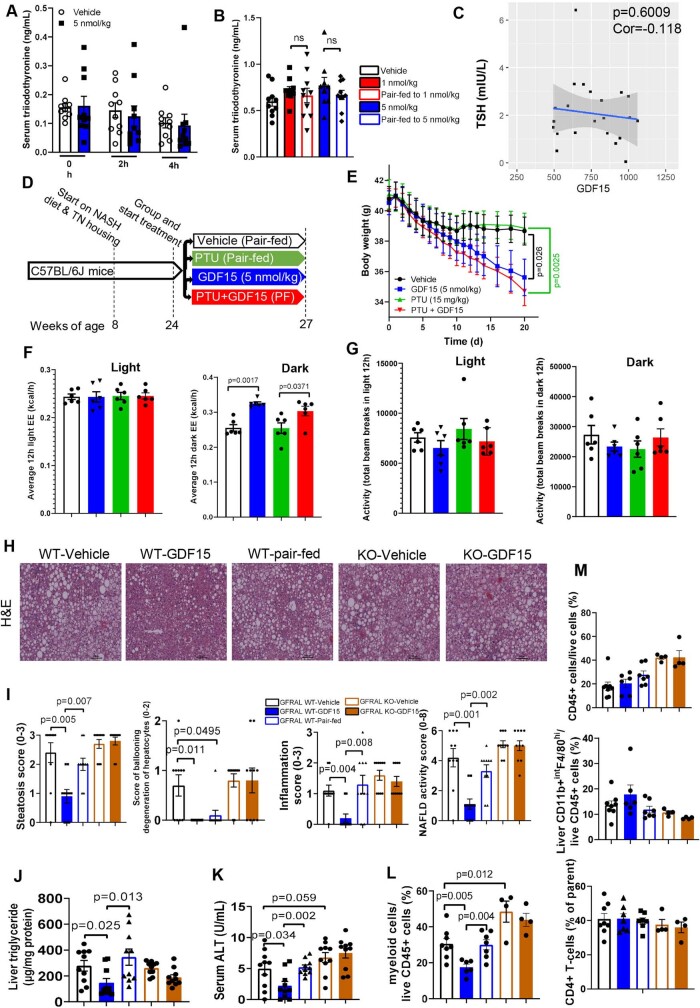
(A-G) Triiodothyronine (T3) is unlikely the primary mechanism contributing to increases in the body weight loss and energy expenditure (EE) induced by GDF15. **A**, A single dose of GDF15 did not change serum T3. Data are means ± SEM, n = 10 mice/group. **B**, GDF15 treatment for 6 weeks did not change serum T3. Data are means ± SEM, n = 10 mice/group except GDF15 (5 nmol/kg) and pair-fed groups, n = 9 mice. Statistical analysis was performed by one-way ANOVA with Tukey’s multiple comparisons test. **C**, Correlation between GDF15 and thyroid-stimulating hormone (TSH) in human plasma (n = 22 participants). The correlation analysis was performed using Pearson’s product-moment correlation (two-sided without adjustment). **D**, Experimental scheme for examining the effects of the T3 blocker propylthiouracil (PTU) and GDF15 on body mass and EE in mice fed with western diet and housed at thermoneutrality. **E**, Body weight. Data are means ± SEM, n = 6 mice/group. P values by two-way ANOVA with Tukey’s multiple comparisons test. **F**, Average 12h-ligh/dark EE. Data are means ± SEM, n = 6 mice/group. P values by one-way ANOVA with Tukey’s multiple comparisons test. **G**, Animal activity during 12h-light/dark circle. Data are means ± SEM, n = 6 mice/group. **(H-M) GDF15 reduces NAFLD and liver inflammation through GFRAL**. **H**, Representative images of paraffin-embedded liver sections stained with H&E. **I**, Steatosis score, score of ballooning degeneration of hepatocytes, inflammation score, and NAFLD activity score. Data are means ± SEM, n = 10 mice/group. P values by unpaired Mann-Whitney test (two-sided). **J**, Liver triglycerides (TG). Data are means ± SEM, n = 10 mice/group. P values by one-way ANOVA with Dunnett’s multiple comparisons test. **K**, Serum alanine aminotransferase (ALT). Data are means ± SEM, n = 10 mice/group. P values by one-way ANOVA with Dunnett’s multiple comparisons test. **L**, Myeloid cell percentage in the liver. Data are means ± SEM, n = 8 mice (WT-vehicle), n = 6 mice (WT-GDF15), n = 7 mice (WT-pair-fed), n = 4 mice (KO-GDF15 and KO-pair-fed). P values by one-way ANOVA with Tukey’s multiple comparisons test. **M**, Flowcytometry analysis for immune cell populations, including: CD45+ cell percentage, liver macrophage percentage, and CD4+ T cells percentage in the liver. Data are presented as means ± SEM. Data are means ± SEM, n = 8 mice (WT-vehicle), n = 6 mice (WT-GDF15), n = 7 mice (WT-pair-fed), n = 4 mice (KO-GDF15 and KO-pair-fed). Source data

Another important mechanism contributing to reductions in energy expenditure with caloric restriction involves the thyroid hormone triiodothyronine^29^. GDF15 is known to activate the hypothalamic–pituitary axis^30^, suggesting this may be important for maintaining energy expenditure. However, the levels of thyroid-stimulating hormone (TSH) were not altered with GDF15 treatment in mice (Extended Data Fig. 5a,b) and chronic treatment of mice with the triiodothyronine blocker propylthiouracil did not prevent GDF15-induced weight loss or increased energy expenditure (Extended Data Fig. 5d–g) compared with pair-feeding. Similarly, there was also no correlation between TSH and GDF15 in women with obesity who were previously enrolled in a diet-induced weight loss program^31^ (Extended Data Fig. 5c). Thus, GDF15 maintains energy expenditure compared to caloric restriction independent of housing temperature, time of feeding or thyroid hormone.

## GDF15 maintains energy expenditure through GFRAL

In mice housed at 21 °C and fed a high-fat diet, *Gfral*-knockout (KO) mice are more obese than wild-type (WT) controls and are resistant to the appetite-suppressing effects of GDF15 over 7–10 days^4–7^, but whether this receptor is also important for the effects of GDF15 to reduce body mass and NASH at thermoneutrality over a more prolonged treatment period is not known. Using similar treatment conditions as described above (Fig. 2f), we found that *Gfral*-KO mice^32^ had greater food intake and were resistant to the appetite-suppressing effects of GDF15 (1 nmol per kg) compared with their WT littermates (Fig. 2g). *Gfral*-KO mice had a greater body mass compared with their WT littermates (Fig. 2h). In WT mice, GDF15 reduced body mass compared with WT vehicle-treated and pair-fed controls—effects that were attenuated in *Gfral*-KO mice (Fig. 2h). *Gfral*-KO mice were also resistant to the effects of GDF15 to lower steatosis, ballooning, inflammation and NAFLD activity scores (Extended Data Fig. 5h,i), liver triglycerides (Extended Data Fig. 5j) and serum ALT (Extended Data Fig. 5k). GDF15 may exhibit anti-inflammatory effects in the liver by acting on myeloid cells independently of GFRAL^33^; however, we found that, consistent with liver inflammation scoring, GDF15 reduced liver myeloid cell populations through GFRAL and independently of reductions in food intake (Extended Data Fig. 5l,m and Supplementary Fig. 2). The data indicate that, consistent with changes in body mass, GDF15 reduces NAFLD and liver inflammation through a GFRAL-dependent mechanism that is independent of reductions in food intake.

**Extended Data Fig. 6:**
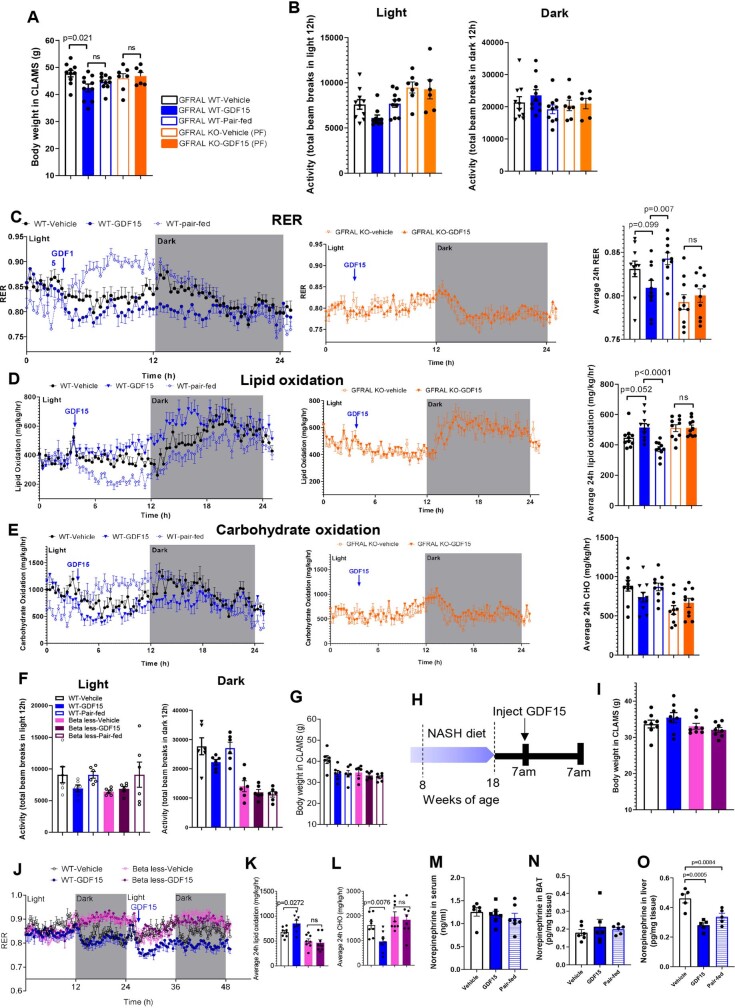
GDF15 increases energy expenditure and fatty acid oxidation through GFRAL and beta-adrenergic signalling. **A**, Body weight in Comprehensive Lab Animal Monitor System (CLAMS). Data are means ± SEM, n = 10 mice (WT-vehicle, WT-GDF15 and WT-pair-fed), n = 7 mice (KO-Pair-fed) and n = 6 mice (KO-GDF15). P values by one-way ANOVA with Dunnett’s multiple comparisons test. **B**, Animal activity during 12h-light/dark circle. Data are means ± SEM, n = 10 mice (WT-vehicle, WT-GDF15 and WT-pair-fed), n = 7 mice (KO-Pair-fed) and n = 6 mice (KO-GDF15). **C**, Average respiratory exchange ratios (RER) curves in WT and GFRAL KO mice and quantification of average 24h RER. Data are means ± SEM, n = 10 mice/group. P values by one-way ANOVA with Šídák’s multiple comparisons test. **D**, Average lipid oxidation curves in WT and GFRAL KO mice and quantification of average 24h lipid oxidation. Data are means ± SEM, n = 10 mice/group. P values by one-way ANOVA with Šídák’s multiple comparisons test. **E**, Average carbohydrate oxidation (CHO) curves in WT and GFRAL KO mice and quantification of average 24h CHO. Data are means ± SEM, n = 10 mice/group. P values by one-way ANOVA with Šídák’s multiple comparisons test. **F**, Animal activity during 12h-light/dark circle. Data are means ± SEM, n = 6 mice/group. **G**, Body mass of mice in CLAMS. n = 6 mice/group. **H**, Experimental scheme for the effects of a single injection of GDF15 on respiratory exchange ratios (RER), lipid oxidation and carbohydrate oxidation (CHO) in WT and beta-less mice. **I**, Body weight in CLAMS. Data are means ± SEM, n = 8 mice/group. **J**, Respiratory exchange ratio (RER) over time in WT and beta-less mice. Data are means ± SEM, n = 8 mice/group. **K**, Average fatty acid oxidation over 24hours. Data are means ± SEM, n = 8 mice/group. P values by one-way ANOVA with Tukey’s multiple comparisons test. **L**, Average carbohydrate oxidation over 24h. Data are means ± SEM, n = 8 mice/group. P values by one-way ANOVA with Tukey’s multiple comparisons test. **M**, GDF15 did not change serum norepinephrine. Data are means ± SEM, n = 7 mice/group except vehicle group, n = 6 mice. **N**, GDF15 did not change norepinephrine in the brown adipose tissue (BAT). Data are means ± SEM, n = 6 mice/group. **O**, GDF15 did not change norepinephrine in the liver. Data are means ± SEM, n = 5 mice/group. P values by one-way ANOVA with Tukey’s multiple comparisons test. Source data

To examine whether GDF15 maintains energy expenditure through GFRAL, we conducted studies in WT and *Gfral*-KO mice after 19 days of GDF15 treatment (Fig. 2i)—a timepoint before there were significant differences in body mass between the groups (Fig. 2h and Extended Data Fig. 6a). There was no difference in physical activity between the treatment groups (Extended Data Fig. 6b). Compared with WT vehicle-treated mice, during the dark cycle, GDF15 maintained energy expenditure compared with pair-fed controls, effects that were eliminated in *Gfral*-KO mice (Fig. 2j). This maintenance of energy expenditure with GDF15 compared with pair-fed or *Gfral*-KO mice persisted after correcting for body mass by ANCOVA (Fig. 2k). GDF15 also reduced the respiratory exchange ratio (RER), indicative of higher fatty acid and lower carbohydrate oxidation compared with the pair-fed controls—an effect that was also eliminated in *Gfral*-KO mice (Extended Data Fig. 6c–e). Thus, consistent with greater long-term weight loss, GDF15 increases fatty acid oxidation and prevents reductions in energy expenditure elicited by caloric restriction through a mechanism requiring GFRAL.

## GDF15 increases β-adrenergic signalling

**Fig. 3:**
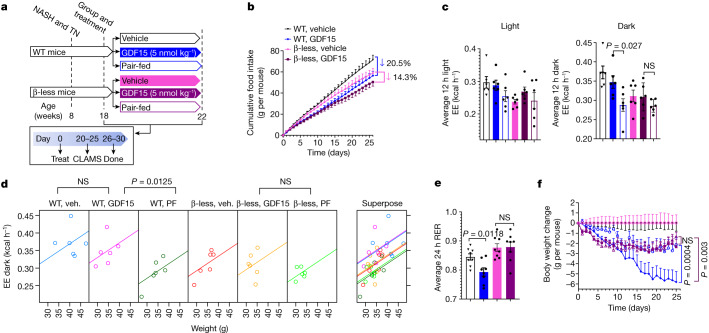
GDF15 increases energy expenditure and fatty acid oxidation through β-adrenergic receptors. **a**, Experimental schematic for the effects of GDF15 and matched caloric restriction in WT and β-less mice. **b**, Cumulative food intake. Data are mean ± s.e.m. *n* = 6 mice per group. *P* values were calculated using two-way ANOVA with Tukey’s multiple-comparison test. **c**, The average energy expenditure during a 12 h–12 h light–dark cycle. Data are mean ± s.e.m. *n* = 6 mice per group. *P* values were calculated using one-way ANOVA with Šidák’s multiple-comparison test. **d**, ANCOVA of energy expenditure against body weight of mice using body mass as a covariate and treatment as a fixed factor (two-sided without adjustment). *n* = 6 mice per group. Veh., vehicle. **e**, Average RER over 24 h. Data are mean ± s.e.m. *n* = 8 mice per group. *P* values were calculated using one-way ANOVA with Tukey’s multiple-comparison test. **f**, Body weight change (in grams) over time. Data are mean ± s.e.m. *n* = 6 mice per group. *P* values were calculated using two-way ANOVA with Tukey’s multiple-comparison test. Source data

GFRAL is exclusively expressed in the hindbrain^34^, suggesting that GDF15 probably increased energy expenditure through a brain–somatic tissue circuit. In addition to changes in triiodothyronine, the suppression of sympathetic nervous system (SNS) activity is an important factor contributing to reductions in energy expenditure with caloric restriction and weight loss^35,36^. To test whether GDF15 treatment may promote weight loss compared with pair-fed controls through increased SNS activity, we administrated GDF15 to WT mice and mice lacking β_1_, β_2_ and β_3_ adrenergic receptors (hereafter, β-less mice) (Fig. 3a). The β-less mice responded normally to the effects of GDF15 to suppress food intake (Fig. 3b); however, when placed in metabolic cages before differences in body mass occurred (Extended Data Fig. 6g), β-less mice were resistant to the effects of GDF15 to maintain energy expenditure during the dark cycle compared to WT controls (Fig. 3c,d and Extended Data Fig. 6f). The β-less mice were also resistant to the effects of GDF15 to reduce RER and increase fatty acid oxidation (Fig. 3e and Extended Data Fig. 6h–l). Furthermore, similar to observations in *Gfral*-KO mice, chronic treatment of β-less mice with GDF15 did not reduce body mass more than pair-feeding alone (Fig. 3f). Taken together, these data indicate that GDF15 promotes weight loss by increasing energy expenditure through a GFRAL–β-adrenergic signalling axis.

**Extended Data Fig. 7:**
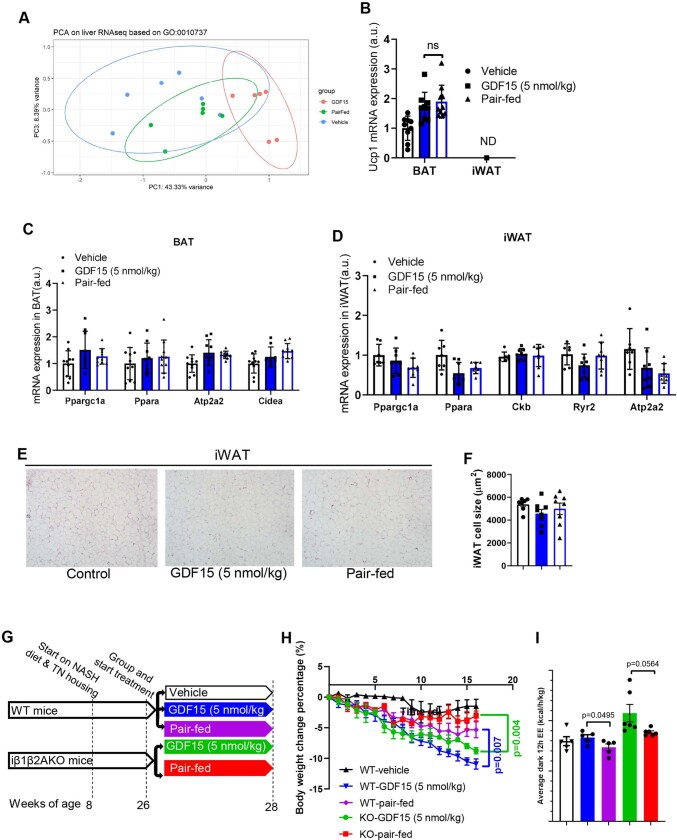
GDF15 does not alter SNS signalling in liver, expression of thermogenic genes in adipose tissue and does not require adipose tissue AMPK to induce weight loss. **A**, Principal component analysis (PCA) for liver RNAseq data based on protein kinase A signalling pathway (GO: 0010737), n = 6 mice/group. **B**, *Ucp1* gene expression in iWAT and iBAT. Data are means ± SEM, n = 9 mice/group except pair-fed group, n = 10 mice/group. Statistical analysis was performed by one-way ANOVA with Tukey’s multiple comparisons test. **C**, Gene expression in iBAT. Data are means ± SEM, n = 10 mice/group except GDF15 group, n = 9 mice. **D**, Gene expression in iWAT. Data are means ± SEM, n = 8 mice/group except vehicle group, n = 7 mice. **E**, Representative images of paraffin-embedded iWAT sections stained with H&E. **F**, Mean cell size of iWAT. Data are means ± SEM, n = 8 mice/group except vehicle group, n = 7 mice. **G**, Experimental scheme for the effect of GDF15 on body mass and energy expenditure in WT and an inducible model for deletion of the AMPKβ1 and β2 subunits in adipocytes (iβ1β2AKO) mice fed with western diet and housed at thermoneutrality. **H**, Body weight change. Data are means ± SEM, n = 5 mice/group (WT mice), n = 6 mice/group (iβ1β2AKO mice). P values by two-way ANOVA with Tukey’s multiple comparisons test. **I**, Average 12h-dark EE. Data are means ± SEM, n = 5 mice/group (WT mice), n = 6 mice/group (iβ1β2AKO mice). P values by one-way ANOVA with Tukey’s multiple comparisons test. Source data

**Extended Data Fig. 8:**
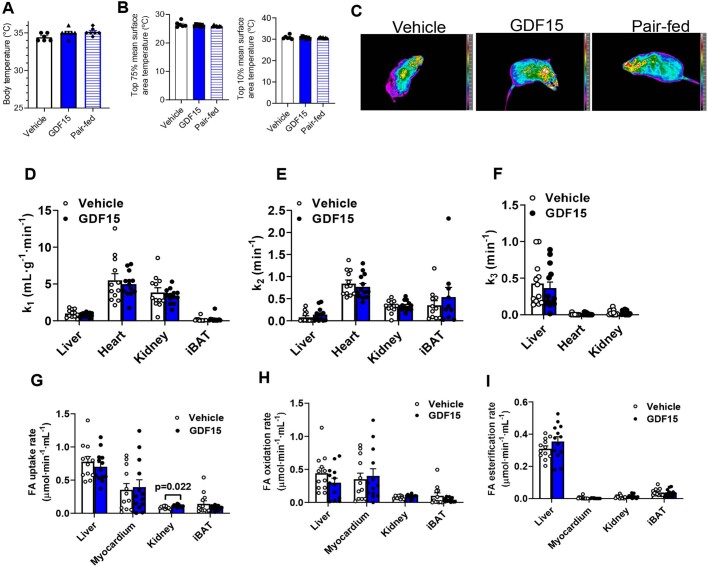
GDF15 does not alter body temperature or oxidative metabolism in liver or brown adipose tissue. **A**, Body temperature (rectal temperature). Data are means ± SEM, n = 6 mice/group (Vehicle), n = 7 mice/group (GDF15 and Pair-fed groups). **B**, Surface temperature of mice. Data are means ± SEM, n = 6 mice/group (Vehicle), n = 7 mice (GDF15 and Pair-fed groups). **C**, Representative infrared images of mice. Images are displayed using the rainbow high contrast colour palette in the FLiR Research IR program. Data are means ± SEM, n = 6 mice/group (Vehicle and GDF15 groups), n = 7 mice (Pair-fed). **D**, k_1_ in mL·g^−1^·min^−1^ (Tissue blood flow index based on the uptake rate of [^11^C]-acetate) in liver, interscapular brown adipose tissue (iBAT), heart and kidney. Data are means ± SEM, n = 12 mice/group (Vehicle), n = 13 mice/group (GDF15). **E**, k_2_ in min^−1^ (oxidative metabolism index: the rapid fractional tissue clearance of [^11^C]-acetate) in liver, iBAT, heart and kidney. Data are means ± SEM, n = 12 mice/group (Vehicle except iBAT, n = 11 mice), n = 11 mice, n = 13 mice/group (GDF15 except iBAT, n = 11 mice). **F**, k_3_ in min^−1^ (non-oxidative disposal: trapping of ^11^C in tissue as free [^11^C]-acetate or other metabolites, such as lipids) in liver, heart and kidney. Data are means ± SEM, n = 12 mice/group (Vehicle), n = 13 mice/group (GDF15). **G**, Fatty acid (FA) uptake rate in liver, iBAT, myocardium and kidney using [^11^C]-palmitate PET. Data are means ± SEM, n = 12 mice (Vehicle), n = 13 mice (GDF15). P values by Paired t test **H**, FA oxidation rate in liver, iBAT, myocardium and kidney. Data are means ± SEM, n = 12 mice (Vehicle), n = 13 mice (GDF15). **I**, FA esterification rate in liver, iBAT, myocardium and kidney. Data are means ± SEM, n = 12 mice (Vehicle), n = 13 mice (GDF15). Source data

**Extended Data Fig. 9:**
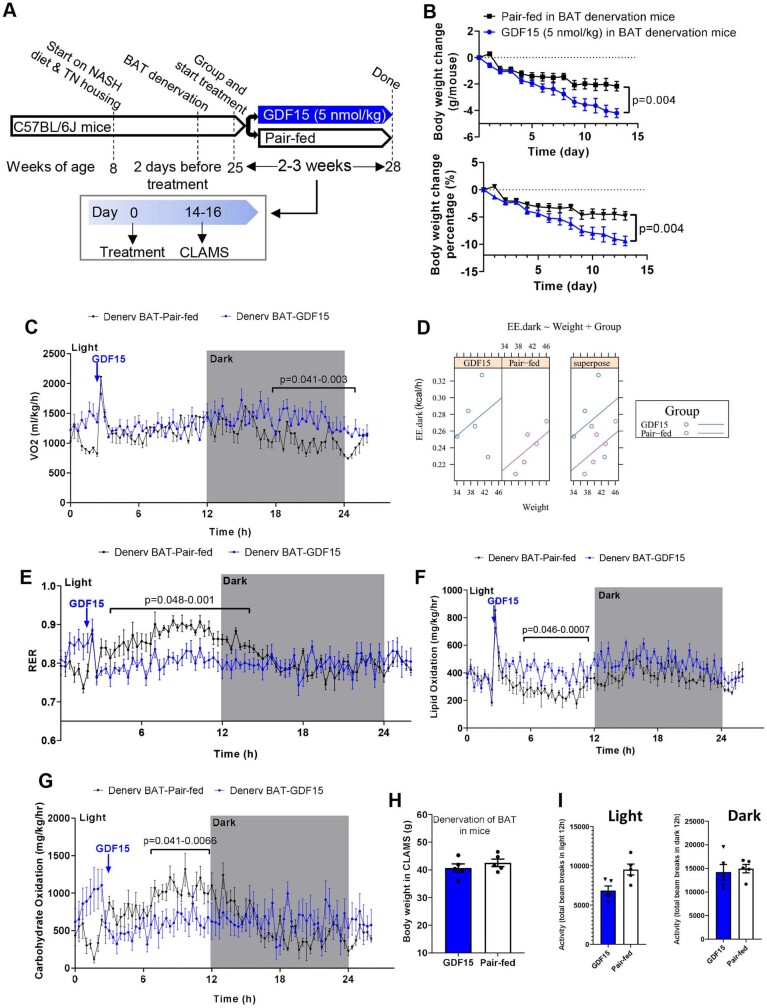
Denervation of BAT does not prevent GDF15-induced body weight loss, energy expenditure and fatty acid oxidation. **A**, Experimental scheme for testing the effects of GDF15 and matched caloric restriction in iBAT denervated C57BL/6J mice fed a western diet and housed at thermoneutrality (TN, 29 °C). The BAT of mice were denervated by 6-hydroxydopamine hydrobromide (6OHDA). **B**, Body weight change and percentage body weight change. Data are means ± SEM, n = 5 mice/group. P values by unpaired t test (two-sided). **C**, Oxidation consumption curves. Data are means ± SEM, n = 5 mice/group. P values by unpaired t test (two-sided). **D**, ANCOVA of energy expenditure against body weight of mice using body mass as a covariate and treatment as a fixed factor. **E**, Average respiratory exchange ratios (RER) curves. Data are means ± SEM, n = 5 mice/group. P values by unpaired t test (two-sided). **F**, Average lipid oxidation curves. Data are means ± SEM, n = 5 mice/group. P values by unpaired t test (two-sided). **G**, Average carbohydrate oxidation (CHO) curves. Data are means ± SEM, n = 5 mice/group. P values by t test (two-sided). **H**, Body weight. Data are means ± SEM, n = 5 mice/group. **I**, Animal activity during 12h-light/dark circle. Data are means ± SEM, n = 5 mice/group. Source data

To examine what tissues might be contributing to this effect, we measured serum and tissue noradrenaline 2 h after the injection of GDF15 (1 nmol per kg) in mice treated for 30 days. GDF15 did not increase noradrenaline in the serum, intrascapular brown adipose tissue (iBAT) or liver (Extended Data Fig. 6m–o). Consistent with this finding, liver, white adipose tissue and BAT showed no changes in β-adrenergic or futile signalling pathways (Extended Data Fig. 7a–d) and there were no signs of adipose tissue browning (Extended Data Fig. 7e,f). We also found that AMPK adipose tissue-null mice, which are insensitive to β-adrenergic induced increases in adipose tissue thermogenesis^37^, lost comparable body mass to WT mice treated with GDF15 (Extended Data Fig. 7g–i). GDF15 did not change rectal or iBAT temperature (Extended Data Fig. 8a–c). Similarly, GDF15 did not significantly alter oxidative metabolism assessed using positron emission tomography–computed tomography (PET–CT) within the iBAT, heart, liver or kidney compared with the vehicle controls (Extended Data Fig. 8d–i). Oxidative metabolism was below the limits of detection of PET–CT in white adipose tissue or skeletal muscle. Finally, we denervated BAT with a local injection of 6-hydroxydopaminehydrobromide (6OHDA) into the iBAT depot and found that, although this blunted the effects of the β_3_ agonist CL-316,243 to increase energy expenditure and iBAT temperature compared with saline injection (Supplementary Fig. 3), it did not inhibit the ability of GDF15 to promote weight loss, increase energy expenditure or fatty acid oxidation compared with pair-fed controls (Extended Data Fig. 9). Collectively, these data indicate that GDF15 is unlikely to stimulate energy expenditure through β-adrenergic signalling in adipose tissue.

## GDF15 increases calcium cycling in muscle

**Fig. 4:**
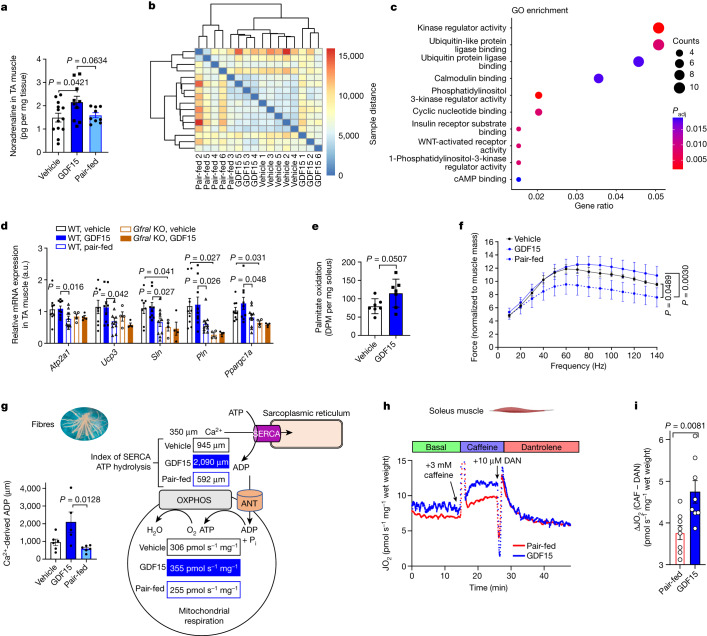
GDF15 increases calcium futile cycling in skeletal muscle. **a**, GDF15 increases noradrenaline in tibialis anterior (TA) muscle. Data are mean ± s.e.m. *n* = 13 (vehicle), *n* = 10 (GDF15), *n* = 9 (pair-fed) mice. *P* values were calculated using one-way ANOVA with Tukey’s multiple-comparison test. **b**, Heatmap of the sample-to-sample distances for TA muscle. *n* = 5 mice per group, except for the GDF15 group, for which *n* = 6 mice. **c**, GO annotation between GDF15 and pair-fed groups. The adjusted *P* value (*P*_adj_) was calculated using the Benjamini–Hochberg method. **d**, Relative gene expression in muscle. Data are mean ± s.e.m. *n* = 9 (WT, vehicle; and WT, GDF15), *n* = 10 (WT, pair-fed) and *n* = 4 (KO, pair-fed; and KO, GDF15) mice. *P* values were calculated using two-way ANOVA with Tukey’s multiple-comparison test. a.u., arbitrary units. **e**, Fatty acid oxidation in soleus muscle. Data are mean ± s.e.m. *n* = 7 mice per group. *P* values were calculated using two-sided unpaired *t*-tests. DPM, disintegrations per minute. **f**, Force–frequency curve of EDL muscles. Data are mean ± s.e.m. *n* = 4 mice per group. *P* values were calculated using two-way ANOVA with Tukey’s multiple-comparison test. **g**, Calculated calcium (Ca^2+^)-derived ADP in permeabilized fibres from red skeletal muscle. Data are mean ± s.e.m. *n* = 6 mice per group, except for the GDF15 group, for which *n* = 5 mice. *P* values were calculated using one-way ANOVA with Tukey’s multiple-comparison test (Supplementary Fig. 5). Schematic of the SERCA efficiency. P_i_, inorganic phosphate. **h**, Real-time trace from a single soleus respiration experiment (*n* = 1 mouse per group) showing the experimental protocol to measure basal, caffeine-stimulated (CAF; 3 mM) and Ca^2+^-independent (10 μM dantrolene (DAN)) respiration (JO_2_). **I**, Ca^2+^-dependent JO_2_ (CAF-DAN) calculated from Extended Data Fig. 10o. Data are mean ± s.e.m. *n* = 8 mice per group. *P* values were calculated using two-sided unpaired *t*-tests. Source data

**Extended Data Fig. 10:**
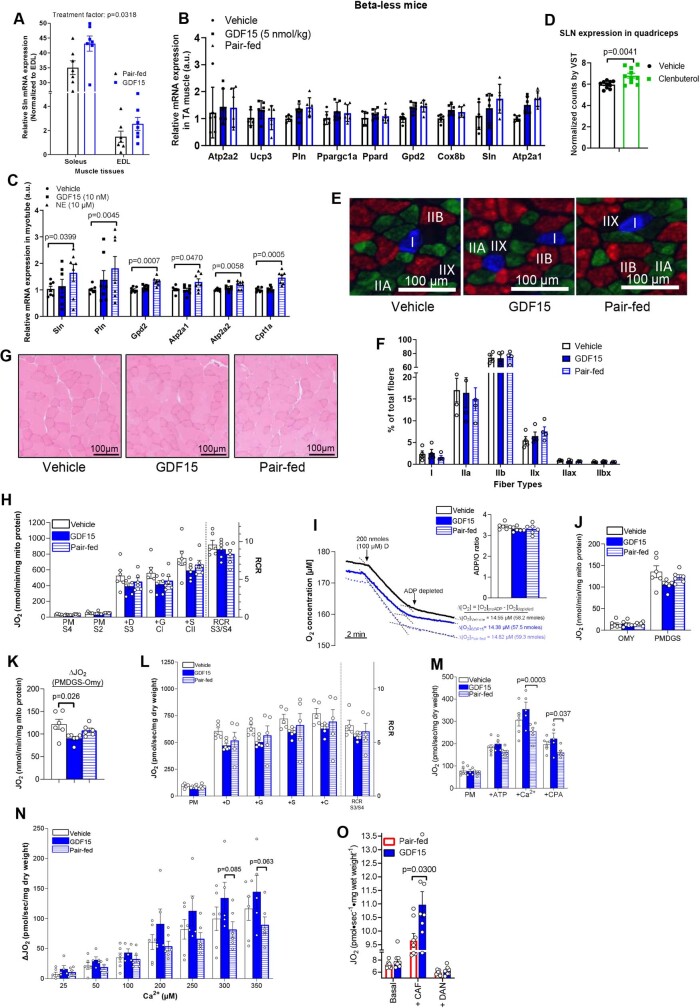
GDF15 increases beta-adrenergic signalling and mitochondrial respiration in skeletal muscle without change fibre type percentages and muscle structure. **A**, Relative *Sln* gene expression level in soleus and extensor digitorum longus (EDL) muscle. Data are means ± SEM, n = 7 mice/group. P values of treatment factor was analysed by Mixed-effects model (REML, two-sided without adjustment). **B**, Relative expression level of *Atp2a1*, *Atp2a2*, *Ucp3, Sln, Pln, Ppard, Gpd2, Cox8b* and *Pppargc1a* in TA muscle from beta-less mice. n = 6 mice/group. **C**, Relative expression level of *Atp2a1*, *Atp2a2*, *Sln, Pln, Gpd2*, and *Cpt1a* in myotubes treated with vehicle, GDF15 and norepinephrine (NE). n = 7 biologically independent samples/group. P values by two-way ANOVA with Tukey’s multiple comparisons test. **D**, *Sln* expression in quadriceps isolated from mice treated with the vehicle or beta-2 agonist (Clenbuterol). Data are means ± SEM, n = 10 mice/group. P values by unpaired t test (two-sided). **E**, Representative immunohistochemical staining for fibre types in gastrocnemius. **F**, Muscle fibre type percentage. Data are mean ± SEM, n = 4 group. **G**, Representative images of frozen gastrocnemius muscle sections stained with H&E (4 mice/group). Within each gastrocnemius, the entire muscle cross-section was visualized and imaged to evaluate the whole cross-section in its entirety. **H**, Absolute mitochondrial respiration (JO_2_); S4: State 4 (pyruvate + malate), S2: State 2 (pyruvate + malate, depleted ADP), S3: State 3 (PMD (+max ADP)), CI: maximal CI respiration (PMDG (+max glutamate)), CII: maximal CII respiration (+max succinate), RCR: S3/S4. Data are means ± SEM, n = 6 mice/group. **I**, Representative trace of oxygen depletion in mitochondria in the presence of 100 μM (200 nmoles) ADP for determination of ADP/O ratios. Data are means ± SEM, n = 6 mice/group. **J**, Absolute mitochondrial JO_2_ with oligomycin and maximal CII supported respiration. OMY: JO_2_ with mitochondria + oligomycin (no substrates), CII: maximal respiration with PMDGS. Data are means ± SEM, n = 6 mice/group. **K**, Change in mitochondrial JO_2_ from OMY with PMDGS. Data are means ± SEM, n = 6 mice/group. P values by one-way ANOVA with Tukey’s multiple comparisons test. **L**, JO_2_ in permeabilized fibres in the presence and absence of ADP and various substrates. Data are means ± SEM, n = 5 mice/group except GDF15 group, n = 6 mice. **M**, Oxygen consumption (JO2) in permeabilized muscle fibres treated with (PM, Pyruvate + Malate, ATP, Ca^2+^ or CPA, cyclopiazonic acid). Data are means ± SEM, n = 6 mice/group except GDF15 group, n = 5 mice. P values by two-way ANOVA with Šídák’s multiple comparisons test. **N**, Change in JO_2_ during a calcium titration in permeabilized fibres. Data are means ± SEM, n = 6 mice/group except GDF15 group, n = 5 mice. P values by two-way ANOVA with Tukey’s multiple comparisons test. P- pyruvate; M - malate; D - ADP; G - glutamate; S - succinate; RCR - respiratory control ratio. **O**, Absolute JO2. Data are means ± SEM, n = 8 mice/group. P values by unpaired t test (two-sided). Source data

β-Adrenergic signalling also increases futile cycling in skeletal muscle by increasing the expression of mitochondrial uncoupling proteins and sarcolipin (SLN)^38–40^. SLN binds to the sarco/endoplasmic reticulum calcium ATPase (SERCA), uncoupling ATPase activity from Ca^2+^ transport, promoting ATP hydrolysis and therefore futile cycling in the presence of increased Ca^2+^ (refs. ^41,42^). In contrast to BAT or liver, we found that noradrenaline levels were elevated in tibialis anterior muscle of GDF15-treated mice (Fig. 4a). Heat maps of the same-to-sample differences from RNA-seq data in tibialis anterior muscle found that vehicle and GDF15-treated animals clustered together distinctly from pair-fed controls (Fig. 4b), a finding distinct from the liver where transcriptomic profiles between pair-fed and vehicle controls were not different (Fig. 1i,j and Extended Data Fig. 7a). Consistent with increases in noradrenaline, Gene Ontology (GO) annotation of differentially expressed genes in skeletal muscle between the GDF15 and pair-fed groups showed that GDF15 increases cAMP/PKA signalling compared with in the pair-fed controls^43^ (Fig. 4c). Targeted analysis revealed higher levels of PKA-regulated genes, including *Ppargc1a*, *Ucp3*, *Atp2a1* (encoding SERCA1), *Sln* and *Pln* compared with pair-fed control or *Gfral*-KO mice, suggesting that endogenous levels of GDF15 may also be important for maintaining this pathway (Fig. 4d). GDF15 treatment also increased *Sln* expression in the oxidative soleus muscle and glycolytic extensor digitorium longus muscles compared with the pair-fed controls (Extended Data Fig. 10a). Consistent with GFRAL not being expressed in the muscle and the response being mediated through the SNS, treatment of β-less mice with GDF15 did not alter muscle gene expression profiles (Extended Data Fig. 10b). Similarly, noradrenaline but not GDF15, increased the expression *Atp2a1*, *Sln* and *Pln* in cultured C2C12 myotubes (Extended Data Fig. 10c). Lastly, analysis of recently published^44^ RNA-seq data of quadriceps muscle from obese mice treated with the β_2_ agonist clenbuterol also found increased *Sln* expression (Extended Data Fig. 10d). Taken together, this suggests that GDF15 enhances genes that are important for futile cycling in skeletal muscle through increases in β-adrenergic signalling.

To examine whether these changes in gene expression induced functional changes in muscle metabolism, we assessed fatty acid oxidation and found that, consistent with in vivo observations of reductions in RER, there was a strong tendency to stimulate fatty acid oxidation in isolated soleus muscle (+44%, *P* = 0.0507; Fig. 4e). Consistent with the more oxidative phenotype, GDF15 treatment improved in situ electrically stimulated contraction of the extensor digitorum longus (EDL) muscle compared with in pair-fed controls (Fig. 4f). These effects were independent of a fibre type transition (Extended Data Fig. 10e,f) or any other overt changes in muscle structure (Extended Data Fig. 10g), suggesting that GDF15 may promote oxidative metabolism. The assessment of oxidative skeletal muscle mitochondrial respiration, by measuring ADP/O ratios or leak respiration in isolated mitochondria (Extended Data Fig. 10h–k) or respiratory control ratios (RCR) in permeabilized muscle fibres (Extended Data Fig. 10l), found that GDF15 did not directly influence mitochondrial coupling efficiency. However, GDF15 increased Ca^2+^-supported respiration in permeabilized muscle fibres compared to vehicle controls (Extended Data Fig. 10m,n). However, based on submaximal ADP responses during an ADP titration (Supplementary Fig. 5), the ADP concentration produced in the presence of 350 µM Ca^2+^ was estimated to reveal that GDF15 treatment elicited greater Ca^2+^-derived ADP compared to both vehicle and pair-fed controls (Fig. 4g and Extended Data Fig. 10m,n). To further examine the importance of calcium futile cycling for driving muscle oxygen consumption, we assessed respiration in the soleus muscle of obese mice fed the NASH diet that were calorically restricted and injected with vehicle (pair-fed) or GDF15 for 21 days. Muscle respiration was assessed basally, in response to caffeine (which increases SERCA activity and intracellular calcium) and after treatment with dantrolene (which inhibits the ryanodine receptor and release of calcium from the sarcoplasmic reticulum). Consistent with our observations in permeabilized fibres, chronic treatment with GDF15 did not significantly increase basal respiration (Fig. 4h,i). As expected, the addition of caffeine stimulated respiration in pair-fed controls; however, in GDF15-treated mice, this response was enhanced by around 30% (Fig. 4h and Extended Data Fig. 10o). Importantly, this increase in respiration was eliminated by the addition of dantrolene (Fig. 4h,i and Extended Data Fig. 10o), indicating that GDF15 enhances skeletal muscle energy expenditure through calcium futile cycling.

## GDF15, muscle thermogenesis and NAFLD in humans

**Extended Data Fig. 11:**
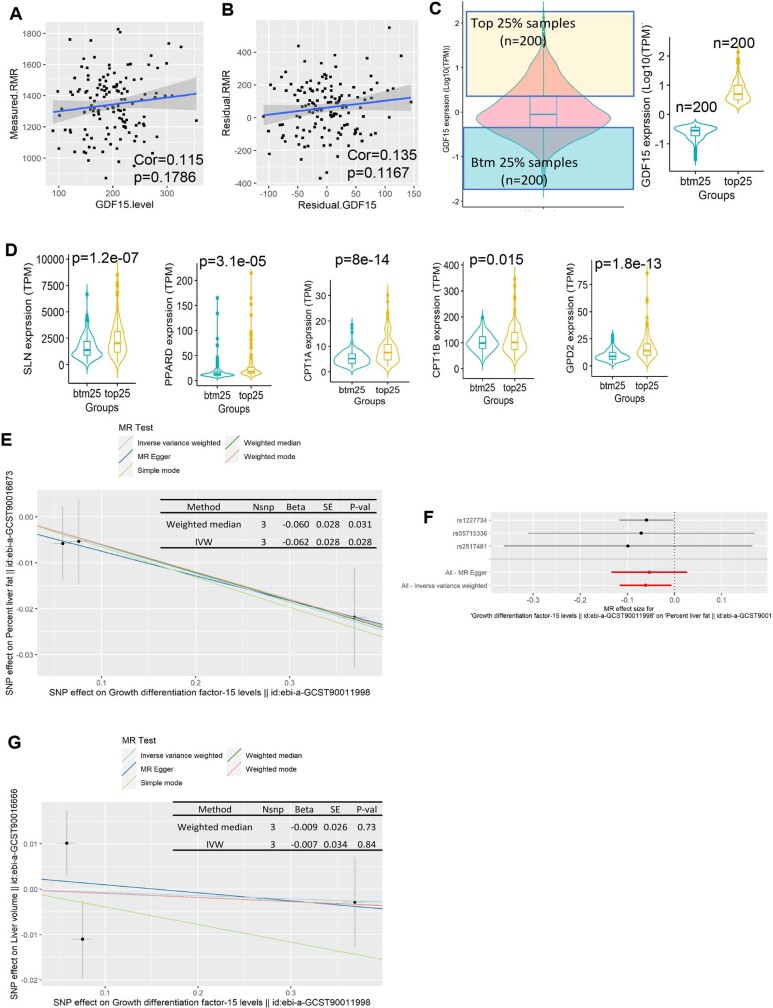
The relationship between GDF15 and resting metabolic rate (RMR) and NAFLD in humans. **A**, Correlation between GDF15 levels and RMR in a population of healthy adults (n = 137 participants). The correlation analysis was performed using Pearson’s product-moment correlation (two-sided). **B**, the correlation between GDF15 levels correcting to weight and mass and RER after correction for fat mass, fat free mass and age from TANITA, by using our published equation Ln BEE = −0.954+0.707 Ln FFM +0.019 Ln FM. The correlation analysis was performed using Pearson’s product-moment correlation (two-sided). **C**, Distribution of GDF15 expression levels (log10(TPM)) from 806 muscle tissues for human subjects in GTEx. The yellow and blue boxes represent the top 25% (n = 200) and bottom 25% (n = 200) groups, respectively. The hinges correspond to first and third quartiles, the whiskers extend to the largest/smallest value, and the centre lines represent the median values. **D**, Relative expression level (TPM) of *SLN, PPARD, CPT1A, CPT1B, GPD2*. n = 200 human subjects per group. P values by unpaired t test (two-sided). The hinges correspond to first and third quartiles, the whiskers extend to the largest/smallest value, and the centre lines represent the median values. **E**, Scatter plot of the SNP-effect on GDF15 and SNP-effect on liver fat percentage in humans by using two sample Mendelian Randomization (2SMR). Error bars indicate 95% CI, n = 32,859 participants in UK Biobank. MR analysis was performed by using Simple median method, MR weighted mode estimator, Weighted median method, MR Egger regression, Inverse variance weighted methods. **F**, Single SNP analysis of GDF15 on liver fat percentage in human, Error bars indicate 95% CI, n = 32,859 participants in UK Biobank. **G**, Scatter plot of the SNP-effect on GDF15 and SNP-effect on liver volume in human. Error bars indicate 95% CI, n = 32,859 participants in UK Biobank. MR analysis was performed by using Simple median method, MR weighted mode estimator, Weighted median method, MR Egger regression, Inverse variance weighted methods. Source data

The studies described above indicate an important role for GDF15 in maintaining energy expenditure elicited through caloric restriction. However, our findings in *Gfral*-KO mice (Fig. 2) indicating reduced energy expenditure and suppression of β-adrenergic signalling pathways in skeletal muscle (Fig. 3) also support a physiological role of endogenous GDF15. We therefore examined these pathways in three distinct clinical populations. First, we assessed circulating GDF15 levels in a population of healthy adults (*n* = 154) in which the resting metabolic rate (RMR) was assessed using a ventilated hood system^45^, and we found that there was a tendency for a weak positive correlation between GDF15 and RMR (Extended Data Fig. 11a) that remained after correction for fat mass, fat-free mass and age (Extended Data Fig. 11b) as we have described previously^46^. However, given RMR was measured in individuals with a stable weight and, by design, in the absence of movement, this finding may not be that surprising given our observations in mice in which the differences with GDF15 were greatest during the dark cycle and after caloric restriction. We therefore analysed RNA-seq data from human skeletal muscle samples (*n* = 806) in the Genotype-Tissue Expression (GTEx) portal and, consistent with our observations in mice, the muscle from participants with greater GDF15 expression (top 25%) had significantly higher *SLN* and other PKA-regulated genes important for fatty acid oxidation compared with those with lower expression of GDF15 (bottom 25%) (Extended Data Fig. 11c,d). Finally, although previous observational epidemiological studies suggested that increases in GDF15 were associated with liver steatosis in humans^47,48^, these studies did not account for confounding factors associated with NAFLD that are known to increase GDF15, such as obesity and mitochondrial stress. Mendelian randomization studies avoid confounding effects that typically affect observational epidemiology studies by randomly assigning groups on the basis of the alleles that they inherit from their parents and associating this genetic variation (that is, *GDF15* single-nucleotide polymorphisms (SNPs)) with the exposure (serum GDF15 protein levels). Thus, to examine the potential translational relevance of our findings in relation to NAFLD, we performed two-sample Mendelian randomization (2SMR) by application of genome-wide association study (GWAS) summary-level data for liver fat content and volume using magnetic resonance imaging (MRI) scans of UK Biobank participants (*n* = 32,859). In contrast to previous epidemiological studies^47,48^, we found that GDF15 was inversely associated with liver fat content (Extended Data Fig. 11e,f) without alterations in liver volume (Extended Data Fig. 11g). These data indicate that GDF15 is associated with increased SLN and reduced NAFLD in clinical populations.

## Discussion

**Extended Data Fig. 12:**
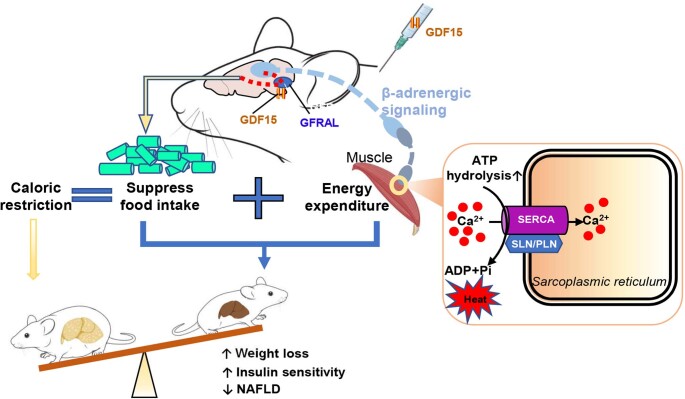
A graphical model depicting the biological circuit linking GDF15 with GFRAL and beta-adrenergic receptors to maintain energy expenditure in muscle during caloric restriction. Mice, liver, muscle and brain illustrations are obtained from BioRender.com (publication license number TZ259QRW6B).

Here, by conducting carefully controlled and extended pair-feeding experiments in mice, we have identified a biological circuit linking GDF15 and GFRAL with β-adrenergic receptors and skeletal muscle calcium cycling (Extended Data Fig. 12). This maintenance of energy expenditure in GDF15-treated mice, compared with calorically restricted pair-fed controls, is associated with increases in skeletal muscle noradrenaline, fatty acid oxidation, oxygen consumption and calcium futile cycling. Notably, this phenocopies mice overexpressing SLN that also lose more body weight and adiposity, have increased energy expenditure and muscle fatty acid oxidation and reduced muscle fatigue compared with pair-fed controls^39,49^. Increased expression of SLN was also observed in the skeletal muscle of people with high levels of GDF15, but there was no significant association between serum GDF15 and RMR in humans. This lack of association between RMR and GDF15 may be expected given our observations of minimal differences in oxygen consumption basally in the absence of increases in calcium cycling as has also been shown in *Sln* transgenic^39^ and KO mice^49^.

It is difficult to precisely quantify the exact contributions of this pathway to basal metabolic rate. It has been estimated that skeletal muscle contributes approximately 30% to total daily energy expenditure^49^ and calcium cycling is approximately 50% of this expenditure^32,50^, meaning that calcium cycling in muscle contributes approximately 15% to whole-body energy expenditure. Given that we observed that GDF15 increased calcium-stimulated respiration compared with in pair-fed control mice by around 30%, this would represent an approximately 5% increase in total energy expenditure—an amount that is comparable to the approximately 100 kcal reduction in energy expenditure observed with modest caloric restriction and weight loss^50^. Although we could not detect any differences in adipose tissue thermogenesis, it is possible that this may also contribute to increases in energy expenditure. Our findings suggest that, given the distinct actions of GDF15 on targeting skeletal muscle thermogenesis, these findings may explain observations of enhanced weight loss when combined with incretin-based therapeutics, which suppress appetite^32^. Future studies investigating the linkages between GDF15–GFRAL signalling, muscle calcium cycling and energy expenditure in humans before and after weight loss will be important to further establish the therapeutic potential of this pathway in adaptive thermogenesis.

## Methods

### Mice

*Gfral*-null mice were generated as described previously^32^ and breeding pairs were provided by R. Seeley. The β-less mice were generated by B. Lowell as described previously^51^ and breeding pairs were provided J. Wu. Studies in mice were carried out at three sites (McMaster University, Université de Sherbrooke and Novo Nordisk). All of the animals used in the study were housed and cared for in accordance with the local guidelines for animal use, and studies were approved by the Animal Ethics Research Board of McMaster University (AUP: 210104), Université de Sherbrooke (2021–3001) and Danish Animal Experiments Inspectorate (2020-15-0201-00756:C01). All of the mice were group-housed on a 12 h–12 h light–dark cycle with ad libitum access to food and water. All of the mice used were males on the C57BL/6J background. All of the mice were housed in either the Solace Zone Heated IVC 32-Cage (Alternative Design Manufacturing & Supply) with HEPA filtered ventilation and temperature-regulated cages kept at 21 or 29 °C or in specific-pathogen free microisolators in a room kept at 21 or 29 °C. Before treatment with GDF15, mice were fed a high-fat, high-fructose diet (NASH Diet: 40 kcal% fat (mostly palm oil), 20 kcal% fructose and 0.02% cholesterol) (Research Diets, D19101102; for experiments performed at McMaster and Sherbrooke University) or western diet (Research Diets, D12079B; for experiments performed at Novo Nordisk). The mice used in chronic experiments at McMaster University were placed on a NASH diet and housed under thermoneutral conditions (~29 °C, 12 h–12 h light–dark cycle) or ambient temperature (~21 °C, 40–60% relative humidity) at 8 weeks of age. Mice at Novo Nordisk were placed on a housing condition (~29–30 °C, 12 h–12 h light–dark cycle) when treatment with GDF15. Before treatment, mice were randomized and separated into different treatment groups matched on body weight and composition, and single-housed. Recombinant GDF15 (Novo Nordisk, 0247-0000-0001; dissolved in synthetic buffer with 5 mM acetate, 240 mM propylene glycol and 0.007% polysorbate 20) was delivered by subcutaneous injection at the start of the light cycle in the morning (07:00–09:00) at 0.3, 1 and/or 5 nmol per kg once daily. The individual dosage was calculated by the body weight 1 day before. Pair-fed animals received the same amount of food as ingested by the corresponding GDF15-treated groups the day before. Body weight and food weight were recorded daily during the treatment period. Food intake was calculated by subtraction of the amount of food content in food hoppers from the amount added the previous day. Spillage and grind of food in cages was carefully monitored every day. Mice were euthanized in a fed state. Terminal blood was collected by cardiac puncture and blood from live animals was collected from a tail snip. Blood samples were centrifuged at 10,000 rpm for 10 min at 4 °C after clotting at room temperature for 30 min, and the supernatant was collected. Serum was saved and stored at −80 °C until use. Mice were anaesthetized using ketamine–xylazine (McMaster) or isoflurane (Novo Nordisk).

### Pharmacokinetic analysis

Mice were placed on a NASH diet and housing condition (29 °C) for 20 weeks starting from 8 weeks of age. After a single subcutaneous injection of recombinant human GDF15 (0.3, 1 and/or 5 nmol per kg) in mice (*n* = 3 per group), tail blood was taken at 0, 0.5, 1, 2, 4, 8 and 24 h to measure recombinant human GDF15 concentrations in the serum using the human GDF-15 DuoSet ELISA kit (R&D Systems, DY957)^52^.

### Body composition measurement

Body composition (lean and fat mass) was analysed using Bruker’s Minispec Whole Body Composition Analyzer (Minispec LF 90II) based on TD-NMR or an MR-scanner from EchoMRITM at the indicated time.

### Measurement of temperature of mice

The core body temperature of the mice was measured by using a digital thermometer to test the rectal temperatures. The surface temperature of the mice was measured by a standardized infrared imaging technique using an infrared camera (T650sc, emissivity of 0.98, FLiR Systems) as described previously^53^.

### Metabolic activity

Metabolic monitoring was conducted using the Comprehensive Laboratory Animal Monitoring System (CLAMS, Columbus Instruments at McMaster) or Promethion system (Sable Systems International, Novo Nordisk). The experiment was conducted after acclimatization to the system for 12 or 24 h. Food intake, physical activity (beam breaks), oxygen consumption (VO_2_), carbon dioxide production (VCO_2_), RER and energy expenditure data were collected every 20 min (CLAMS) or 5 min (Promethion) for the indicated periods. Fatty acid oxidation (mg per kg per h) was calculated using the following equation (1.70 × VO_2_ − 1.69 × VCO_2_). Carbohydrate oxidation (mg per kg per h) was calculated using the following equation (4.58 × VCO_2_ − 3.23 × VO_2_).

### GTT and ITT

Glucose tolerance tests (GTT) and insulin tolerance tests (ITT) were performed 3 and 2 weeks before euthanasia, respectively. Both tests were performed after a 6 h fast. For GTT, mice were injected intraperitoneally with 1.25 g per kg of d-glucose. For ITT, mice were injected intraperitoneally with 1.2 U per kg of insulin (Novorapid). For all tests, blood glucose was measured from a drop of tail blood using the ACCU-CHEK Aviva handheld glucometer (Roche) at 0, 20, 40, 60, 90 and 120 min after injection. Area under the curve analysis was performed using GraphPad Prism (v.9.3.0).

### Biochemical analysis in the serum and liver

Triglycerides (Cayman Chemical, 10010303), non-esterified fatty acids (NEFA) (FUJIFILM Wako Diagnostics, 999-34691, 995-34791, 991-34891, 993-35191), insulin (Crystal Chem, 90080), glucose (FUJIFILM Wako Diagnostics, 997-03001), noradrenaline (Abbexa, abx055012) and ALT (Cohesion Biosciences, CAK1002) were measured according to the kit protocols.

### Histological analysis

Liver and iWAT were collected and fixed with 10% neutral-buffered formalin for 36–48 h. After fixation, the samples were immersed in a 70% alcohol solution. The liver tissues were then processed, paraffin-embedded, serially sectioned and stained with H&E by the McMaster Immunology Research Centre histology Core Facility. Images were taken using the Nikon 90i Eclipse upright microscope. Blinded liver semiquantitative histology scores were assigned to liver sections by a pathologist. Ballooning degeneration of hepatocytes (0–2), steatosis score (0–3) and inflammation score (0–3) were evaluated according to H&E stained liver sections as described previously^54^. NAFLD activity score (0–8) was defined as the sum of these three scores. Quantitative assessment of the size and number of adipocytes in the iWAT was performed using Image J as described previously^55^.

### Single-cell preparations and flow cytometry analysis

For the preparation of liver cells, a lobe of the liver was collected after perfusion of the liver with PBS and digested with enzyme solution buffer containing 0.5 mg ml^−1^ pronase E, 0.088 U ml^−1^ collagenase D and 1% (v/v) DNase I for 30 min at 37 °C. Single-cell suspension of liver non-parenchymal cells was prepared as previously described^56^, with a minor modification. In brief, after digestion, the cells were filtered through a 100 μM cell strainer. After two centrifugation steps of 1 min at 50*g* to remove hepatocytes, the remaining cells in suspension were further filtered through a 40 μM cell strainer. The non-parenchymal single cells were centrifuged at 1,500 rpm for 5 min at 4 °C before proceeding to blocking/antibody staining for flow cytometry. For flow cytometry analysis, the cells were blocked with an antibody against Fc receptors (Fc block (1:200, BD Biosciences, 553142)) and stained for 30 min on ice with an antibody cocktail: CD45.2 BV510 (1:25, BioLegend, 109838), CD11b APC-Cy7 (1:100, Invitrogen, A15390), F4/80-APC (1:100, Invitrogen, 17-4801-82), CD3 BV605 (1:50, BD Biosciences, 563004), CD4 PerCP-Cy5.5 (1:100, BD Biosciences, 550954). 7AAD (1:100, Thermo Fisher Scientific, A1310) was used as a cell viability marker. After the staining, the cells were analysed with a CytoFlex Flow Cytometer (Beckman Coulter Life Sciences). Data analysis was performed using FlowJo (v.10.5).

### RNA isolation, cDNA synthesis and qPCR

Tissues were homogenized and lysed in TRIzol reagent. After centrifuging, the supernatant (aqueous phase) was applied to the RNeasy kit (Qiagen, 74106) for subsequent total RNA extraction and purification according to its protocols. cDNA synthesis was performed using the SuperScript IV Reverse Transcriptase kit (Invitrogen, 18090010) according to the manufacturer’s instructions. The detection of cDNA expression for specific genes was performed by quantitative PCR (qPCR) using the AmpliTaq Gold DNA Polymerase kit (Applied Biosystems, N8080241). Taqman primers were purchased from Thermo Fisher Scientific. Relative mRNA levels were quantified using the Δ*C*_t_ method, using mouse *Actb* (Mm02619580_g1) as an endogenous control. Gene-specific primers were as follows: *Ucp1* (Mm01244861_m1), *Ppara* (Mm00440939_m1), *Ucp3* (Mm01163394_m1), *Ppargc1a* (Mm01208835_m1), *Atp2a1* (Mm01275320_m1), *Atp2a2* (Mm01201431_m1), *Sln* (Mm00481536_m1), *Pln* (Mm04206541_m1), *Ckb* (Mm00834780_g1), *Ryr2* (Mm00465877_m1), *Gpd2* (Mm00439082_m1), *Ppard* (Mm00803184_m1), *Cox8b* (Mm00432648_m1) and *Cidea* (Mm00432554_m1).

### RNA-seq and transcriptomic analysis

Liver and tibialis anterior muscle were collected and snap-frozen in liquid nitrogen before storage at −80 °C. Frozen liver tissues or tibialis anterior muscle (30–50 mg per sample) were homogenized and lysed in TRIzol reagent. After centrifuging, the supernatant (aqueous phase) was applied to the RNeasy kit (Qiagen, 74106) for subsequent total RNA extraction and purification according to the manufacturer’s protocols. All RNA samples passed the BioAnalyzer quality control test. RNA-seq was performed using the Illumina NextSeq 2000 (P2 Flow cell, 2 × 50 bp configuration) system. MultiQC was used for quality control of raw data from RNA-seq^57^. Trim Galore was used to automate quality and adapter trimming as well as quality control. We quantified the expression of transcripts using RNA-seq data through Salmon^58^. Salmon’s transcript-level quantification DESeq2 was used to detect DEGs^59^ using the following threshold: for liver samples, |log_2_[fold change]| > 1, adjusted *P* < 0.05; for tibialis anterior muscle samples: |log_2_[fold change]| > 0.6, adjusted *P* < 0.1. PCA was performed using VST data through DESeq2. Functional enrichment analysis was performed by GO enrichment analysis^60^ and Kyoto Encyclopedia of Genes and Genomes (KEGG) mapping^61^ using the GOstats (https://bioconductor.org) and KEGG.db (https://bioconductor.org) packages, respectively. The results were illustrated in a gene-concept network diagram using the cnetplot package (https://bioconductor.org). Transcriptomic analyses were performed using the Linux system, R and RStudio software. RNA-seq data of quadriceps samples from mice treated with β_2_ agonist clenbuterol were downloaded from the NCBI Sequence Read Archive under reference number PRJNA756816 (ref. ^44^). We quantified the expression of transcripts using RNA-seq data through Salmon^58^. *Sln* expression in the quadriceps was determined using VST data.

### PET–CT Imaging

Male C57Bl/6J mice were placed on a NASH diet and thermoneutral housing conditions (29 °C) for 4 weeks starting from 7 weeks of age. Using a randomized crossover design (*n* = 13), mice were fasted for 7 h and then received a single subcutaneous injection of either a vehicle or GDF15 (5 nmol per kg), 4 h before a sequential dynamic PET acquisition with [^11^C]acetate and [^11^C]palmitate. Experimental sessions were then repeated 7 days later. PET was performed with the avalanche photodiode-based small-animal LabPET8 scanner of the Sherbrooke Molecular Imaging Center (Centre de recherche du CHUS, Université de Sherbrooke). Mice were first anaesthetized (2% isoflurane in 1.5 L min^−1^ of oxygen) then injected intravenously with a 10 MBq bolus of [^11^C]acetate (100 µl final volume in saline solution) through the caudal vein followed by a 15 min list-mode PET acquisition. Then, a bolus of 10 MBq of [^11^C]palmitate (100 µl final volume in saline solution) was injected, and a 15 min list-mode PET acquisition was performed. Residual [^11^C]acetate activity during [^11^C]palmitate acquisition was corrected by acquiring a 60 s frame before the injection of [^11^C]palmitate, accounting for the disintegration rate of [^11^C]. Finally, low-dose CT images were acquired from the integrated X-O small animal CT scanner on the Triumph platform.

For [^11^C]acetate scans, images were reconstructed into 26 dynamic frames (12 × 10, 8 × 30 and 6 × 90 s), whereas [^11^C]palmitate scans were reconstructed into 29 dynamic frames (1 × 60, 12 × 5, 6 × 10, 6 × 30 and 4 × 150 s) using a three-dimensional maximum-likelihood estimation method with 20 iterations, span of 63, field of view of 80 mm with a final matrix resolution of 160 × 160 × 128 and a voxel size of 0.5 × 0.5 × 0.597 mm. For the [^11^C]acetate, input curves were extracted as described previously^62^. In brief, using Amide (v.1.0.4), an image-derived input function (IDIF) was obtained by manually positioning a region of interest (ROI) in the vena cava, above the kidneys and below the myocardial blood pool. The [^11^C]acetate IDIF was then corrected for [^11^C]-labelled metabolites^63^. Tissue ROIs were drawn on the liver, kidneys, myocardium, white adipose tissue, iBAT, quadricep and gastrocnemius muscles. Quantitative data was obtained from the resultant time-activity curves and used to estimate tissue blood flow index (on the basis of the uptake rate of [^11^C]acetate, *k*_1_ in ml g^−1^ min^−1^), oxidative metabolism index (the rapid fractional tissue clearance, *k*_2_ in min^−1^, of [^11^C]acetate) and non-oxidative disposal (trapping of ^11^C in tissue as free [^11^C]acetate or other metabolites, such as lipids, *k*_3_ in min^−1^) using a standard two-compartment, two-tissue, kinetic model^63^. For BAT, a four-compartment, two-tissue, kinetic model was applied, as previously described^64,65^. For the [^11^C]palmitate, IDIFs were obtained as described for [^11^C]acetate and ROIs were drawn on the liver, myocardium, kidneys and iBAT. Fatty acid oxidation, esterification and uptake, and triglyceride release rates were calculated using a three-compartment, two-tissue, kinetic model^66,67^.

### Cell culture of myotubes

C2C12 cell line was purchased from ATCC and authenticated by short tandem repeat profiling at ATCC and tested negative for mycoplasma contamination. C2C12 cells were maintained in Dulbecco’s modified Eagle medium (DMEM) containing 10% fetal bovine serum at 37 °C in 5% CO_2_. After reaching confluence, cells were differentiated to myotubes in DMEM supplemented with 2% horse serum for 5–7 days. Myotubes were treated with vehicle, GDF15 (10 nM) and noradrenaline (10 μM) for 15 h. Myotubes were collected, and RNA isolation, cDNA synthesis and qPCR were performed as described above.

### Ex vivo determination of fatty acid oxidation in soleus muscle

Soleus muscles were carefully dissected tendon to tendon for muscle incubations as described previously^68,69^. Fatty acid metabolism experiments were conducted using procedures previously described^68,69^. In brief, isolated soleus muscles were placed in warmed (30 °C) Krebs–Henseleit buffer pH 7.4 containing 2 mm pyruvate, 4% fatty-acid-free bovine serum albumin and 0.5 mm palmitic acid. After an initial incubation of 15–30 min in a glass vial, the incubation buffer was replaced with Krebs–Henseleit buffer supplemented with 0.5 μCi ml^−1^ [^14^C]palmitate (PerkinElmer, NEC534250UC) for 60 min with vials containing 450 μl of benzethonium hydroxide. Muscles were removed at the end of the chase period and rinsed and frozen under liquid nitrogen for later use. A total of 1 ml of acetic acid was then carefully added to the glass vial, which was immediately sealed. The acetic acid liberates CO_2_ produced by fatty acid oxidation through the TCA cycle. Glass vials were then placed on a shaker at 75 rpm for 1 h to allow for benzethonium hydroxide to trap the released CO_2_. The inner Eppendorf tube containing benzethonium hydroxide was carefully placed into a plastic scintillation vial containing 5 ml of scintillation fluid and allowed to quench overnight in the dark. Half of the soleus muscle were homogenized in 1.5 ml of chloroform:methanol solution (2:1). Then, 2.0 ml of distilled H_2_O was added to the supernatant fraction in the new tube and vortexed gently. The aqueous phase was then transferred to a plastic scintillation vial containing 5 ml of scintillation fluid. DPMs were measured by a scintillation counter (Beckman coulter, LS 6500 multi-purpose scintillation counter). Data are represented as the sum of DPMs from the CO_2_ and acid soluble intermediates and normalized to tissue weight.

### Chemical denervation of iBAT

Denervation of iBAT was achieved by a local injection of 6OHDA (10 mg ml^−1^) in saline containing 1% ascorbic acid into five distinct spots across the iBAT pad (5 μl per spot)^70^. Mice were allowed to recover for 48 h. For the confirmation of BAT denervation, we treated mice with the β_3_ agonist CL-316,243 and measured BAT temperature using a standardized infrared imaging technique using an infrared camera (T650sc, emissivity of 0.98, FLiR Systems)^53^ and energy expenditure in CLAMS^37,71,72^.

### Muscle functional testing

Muscle functional testing was performed in vitro using the horizontal bath of a whole-mouse test system (1300A, Aurora Scientific). Ringer’s solution (120 mM NaCl, 4.7 mM KCl, 2.5 mM CaCl_2_, 1.2 mM KH_2_PO4, 1.2 mM MgSO_4_, 25 mM HEPES, 5.5 mM glucose) within the horizontal bath was bubbled with oxygen for 30 min before experimental initiation. In brief, for muscle functional testing, the EDL muscle was isolated and, using braided silk at both the proximal and distal muscle tendon junctions, secured to both a stationary lever arm hook and a force transducer (model 809c, Aurora Scientific) within the horizontal bath. In this position, the EDL muscle is aligned in parallel between two stimulation electrodes. The EDL muscle was allowed to rest for 10 min before stimulation. To determine the optimal muscle length, the EDL muscle was stimulated at different resting tensions until a maximum twitch tension was determined^73^. The EDL muscle rested for 2 min after this optimization. A force–frequency curve was used to determine peak tetanic force. This force determination consisted of a 1 s stimulation every 30 s beginning at 10 Hz and increasing in stimulation frequency in 10 Hz increments. All data were collected and analysed using Dynamic Muscle Control and Analysis Software (v.615A, Aurora Scientific).

### Immunohistochemical staining of mouse muscle

H&E staining was conducted on frozen gastrocnemius muscle sections using standard protocols. Within each gastrocnemius, the entire muscle cross-section was visualized and imaged to evaluate the whole cross-section in its entirety. Muscle fibre typing was performed as described previously^74^. Immunofluorescence was visualized using the Nikon Eclipse 90i microscope (Nikon) and analysed using NIS-Elements AR software (Nikon, v.5.41.02). To determine fibre-type percentage, a total of 4,000 fibres (types I, IIa, IIb and IIx) were counted per gastrocnemius cross-section (*n* = 4 per group)^75^.

### Respiration in mitochondria isolated from red skeletal muscle

Skeletal muscle mitochondria were isolated using temperature controlled (4 °C) differential centrifugation as previously described^76^. In brief, hindlimb skeletal muscles (red gastrocnemius, plantaris, red tibialis anterior, soleus and red portion of the quadriceps) were excised, trimmed of visible fat and connective tissue, weighed and minced in isolation buffer (100 mM sucrose, 100 mM KCl, 50 mM Tris-HCl, 1 mM KH_2_PO_4_, 0.1 mM EGTA, 0.2% BSA and 1 mM ATP, pH 7.4). Minced tissue was homogenized and centrifuged at 800*g* for 10 min to separate the subsarcolemmal and intermyofibrillar mitochondrial fractions. The pellet containing intermyofibrillar mitochondria was resuspended and treated with a protease subtilisin A (0.025 g per mg wet tissue) for 5 min and ice-cold isolation buffer was subsequently added to stop the protease. The samples were immediately centrifuged at 5,000*g* for 5 min and the pellet was resuspended and centrifuged at 800*g* for 10 min to liberate the intermyofibrillar mitochondria in the supernatant. Subsarcolemmal and intermyofibrillar mitochondria were further centrifuged at 10,000 g for 10 min, resuspended, and combined before centrifugation twice at 10,000*g* for 10 min to recover the final mitochondrial pellet. These pellets were resuspended in Mg^2+^ absent MiR05 (0.5 mM EGTA, 60 mM potassium lactobionate, 10 mM KH_2_PO_4_, 20 mM HEPES, 110 mM sucrose, 1 g l^−1^ fatty acid free BSA, pH 7.2) and kept on ice until respiration experiments were conducted.

Respiration experiments were performed in the Oroboros Oxygraph-2k system at 37 °C with constant stirring. A total of 20 µg of mitochondrial protein was loaded per 2 ml chamber (quantified by Bradford protein assay). ADP/O ratios were calculated using the change in oxygen content (nmol) after the addition of ADP (separately following a 50 µM (100 nmol) bolus and, when depleted, a 100 µM (200 nmol) bolus) in the presence of 5 mM pyruvate and 2 mM malate. Maximal respiration was assessed with subsequent additions of 5 mM ADP, 10 mM glutamate (maximal complex I supported respiration) and 10 mM succinate (maximal complex II supported respiration). RCRs were quantified as the ratio of state 3 (saturating ADP) to state 4 (presence of pyruvate and malate, absence of ADP) respiration. In a separate experiment, 0.5 µM oligomycin was initially added to the chamber and respiration determined in the presence of saturating mixed substrates (pyruvate, malate, ADP, glutamate and succinate).

Mitochondrial purity was checked by western blotting as previously described^77^. In brief, mitochondrial samples were added on top of 1 ml 60% Percoll (1.336 ml 5× SMEA, 4 ml Percoll, 1.334 ml distilled H_2_O; density 1.08–1.12 g ml^−1^). The samples were centrifuged at 20,000*g* for 5 h at 4 °C. The purified mitochondrial layer was removed and suspended in 1 ml isolation buffer and centrifuged at 12,000*g* for 10 min at 4 °C to remove the residual Percoll. The final pellet was resuspended in isolation buffer at stored at −80 °C until the samples were prepared for western blotting. The primary antibodies included COXI (1:500, OXPHOS cocktail, Abcam, Ab110413), COXIV (1:30,000, Invitrogen, A21347), GLUT4 (1:2,500, Abcam, Ab654), calnexin (1:2,000, Sigma-Aldrich, C4731) and SERCA2 (1:1,000, Abcam ab2861).

### Respiration in permeabilized muscle fibres from red skeletal muscle

Permeabilized muscle fibres were prepared from red gastrocnemius muscle as previously described^78,79^. In brief, muscle was placed in ice-cold BIOPS (50 mM MES, 7.23 mM K_2_EGTA, 2.77 mM CaK_2_EGTA, 20 mM imidazole, 0.5 mM dithiothreitol, 20 mM taurine, 5.77 mM ATP, 15 mM PCr and 6.56 mM MgCl_2_·H_2_O, pH 7.1) and fibre bundles were separated with fine-tipped forceps underneath a microscope (MX6 Stereoscope, Zeiss Microsystems). Fibres were incubated in 40 µg ml^−1^ saponin for 30 min and washed in MiR05 respiration buffer (respiration experiments) for 15 min. Mitochondrial respiration experiments were performed in MiR05 respiration buffer in an Oxygraph high-resolution respirometer at 37 °C (Oroboros Instruments) with constant spinning at 750 rpm. Experiments were conducted at room air saturation with reoxygenation after the addition of each substrate (~180–195 µM O_2_). All experiments were performed in the presence of 5 µM blebbistatin, 5 mM pyruvate and 1 mM malate. For submaximal ADP experiments, ADP was titrated at various concentrations (25, 100, 250, 500, 1,000, 2,000, 4,000, 6,000, 8,000, 10,000 µM ADP) followed by the addition of 10 mM glutamate, 10 mM succinate and 10 µM cytochrome *c*. RCRs were calculated by dividing maximal state 3 respiration (presence of ADP) by state 4 respiration (pyruvate + malate, absence of ADP). Ca^2+^ experiments were performed with the addition of 5 mM ATP before titrations of CaCl_2_ (25, 50, 100, 200, 250, 300, 350 µM CaCl_2_) as recently reported^80^. After reaching a plateau, 40 µM CPA was added to inhibit SERCA activity. During the Ca^2+^ titration, SERCA hydrolyses ATP, generating ADP to stimulate respiration. We therefore used the one-phase association curve from the ADP titration to estimate the ADP generated during the Ca^2+^ titration as an index of SERCA efficiency. The regression equations from the ADP titrations were as follows: Vehicle: JO_2_ = 127 + (598 − 127) × (1 − exp(−0.0005292 × [ADP])), GDF15: JO_2_ = 128 + (463 − 128) × (1 − exp(−0.0006502 × [ADP])), pair-fed: JO_2_ = 119 + (590 − 119) × (1 − exp(−0.0005858 × [ADP])). Fibre bundles were recovered from all of the experiments, dried and weighed to normalize respiration to tissue weight (pmol s^−1^ mg^−1^ dry weight).

### Respiration in isolated soleus muscles

Respiration experiments in isolated soleus muscles from mice were performed as previously described^81^ with minor modifications. Soleus muscles were isolated from obese mice fed the NASH diet for more than 13 weeks that were calorically restricted and injected with vehicle (pair-fed) or GDF15 for 21 days. In brief, soleus muscles were excised and placed in a sealed vial containing 2 ml of pre-gassed (95% O_2_, 5% CO_2_) modified Kreb’s Ringer (MKR) buffer at 30 °C (115 mM NaCl, 2.6 mM KCl, 1.2 mM KH_2_PO_4_, 10 mM NaHCO_3_, 10 mM HEPES) supplemented with 4% BSA, 0.5 mM palmitate and 10 mM d-glucose for 1 h. After the 1 h preincubation, soleus muscles were transferred into respirometry systems (Oroboros O2k) containing hyper-oxygenated (starting [O_2_], ~500 μM) supplemented MKR buffer with constant stirring at 30 °C. The rate of oxygen consumption (JO_2_) was determined at the baseline for 20 min before the addition of 3 mM caffeine (Sigma-Aldrich, C0750) to stimulate calcium leak and increase ATP hydrolysis. After 10 min of caffeine-mediated respiration, dantrolene (Sigma-Aldrich, D9175, 10 μM in DMSO) was added to the chamber to assess the effects of inhibiting Ca^2+^ release. All soleus muscles were recovered, trimmed of any remaining tendon/connective tissue, blotted dry and weighed for the normalization of respiration to wet muscle weight. All experiments were determined in duplicate (paired soleus muscles) and technical replicates were averaged for each JO_2_ determination.

### Bioinformatics analysis of the GTEx dataset

We have access to the GTEx Analysis V8. The data used for the analyses described in this Article were obtained from dbGaP accession number phs000424.v8.p2 on 11 May 2022. To study the effect of physiological levels of GDF15 on skeletal muscle in humans, we assessed the raw RNA-seq gene count data from the muscle of 803 individuals. We compared the muscle gene expression by establishing two groups on the basis of the GDF15 expression level in the skeletal muscle using the R and RStudio software.

### Correlation analysis of GDF15 and TSH in human

Blood samples were collected after an overnight fast from women with obesity (*n* = 22)^31^. TSH measurements were conducted by the Ottawa Hospital Laboratory Services. GDF15 levels were analysed using a Human GDF-15 Quantikine ELISA Kit according to the manufacturer’s instructions (R&D Systems, DGD150).

### Correlation study on GDF15 and RMR in humans

The RMR of 154 participants was measured using a ventilated hood^45^ (JAEGER Oxycon Pro, Viasys Healthcare). The measurement was performed after an overnight fast between 08:00 and 10:00. The hood was placed over the head of recumbent subjects. The measurement lasted for 40 min, during when the participants were required to keep still yet remain awake. The mean values of every 10 min were then calculated and the minimum values were used as the RMR of the participants. RMR was adjusted for body composition on the basis of TANITA data using our published equation Natural logarithm (Ln)_BEE (basal expenditure)_ = −0.954 + 0.707 Ln_FFM (fat-free mass)_ + 0.019 Ln_FM (fat mass)_ (ref. ^46^). GDF15 levels in human plasma were tested by using human GDF-15 DuoSet ELISA kit (R&D Systems, DY957)^52^. GDF15 levels were corrected for weight and age by multiple linear regression using R.

### 2SMR using GWAS summary data

2SMR was performed using the exposure and the outcome from two non-overlapping and independent datasets to conduct the summary-level instrument exposure analysis and the instrument–outcome association analysis. 2SMR was performed using the TwoSampleMR R package (v0.5.6)^82^. To verify the causal effect of GDF15 on liver fat in humans, we performed 2SMR using the exposure dataset (GDF15, GWAS ID: ebi-a-GCST90011998, sample size: 21,758)^83,84^ and outcome dataset (liver fat percentage, GWAS ID: ebi-a-GCST90016673, sample size: 32,858)^85,86^. To examine the effect of GDF15 on liver volume in humans, we performed 2SMR using the exposure dataset (GDF15, GWAS ID: ebi-a-GCST90011998, sample size: 21,758) and outcome dataset (liver volume, GWAS ID: ebi-a-GCST90016666, sample size: 32,858)^85,86^. We identified genetic variants (SNPs) associated with blood GDF15 protein levels in the GWAS catalogue dataset based on *cis*-pQTL (within 500 kb of the *Gdf15* gene), and further selected proxy SNPs by linkage disequilibrium (LD)-clumping (p1=5e-08, clump=TRUE, p2 = 1e-07, r2 = 0.001, kb = 10000). After dropping duplicate exposure–outcome summary sets, we further performed sensitivity analyses, including heterogeneity statistics, horizontal pleiotropy and leave-one-out analysis. After confirming that there was no heterogeneity or horizontal pleiotropy, we next performed MR analysis and visualized the results using the scatter plot and forest plot functions in the TwoSampleMR R package. We used MR Steiger directionality test^87^ to evaluate causal direction between GDF15 and liver fat in humans. The inverse variance weighted method was used to assess the significance of the causal effect of the exposure on the outcome. 2SMR was performed using R and RStudio.

### Statistics

Statistical analyses were performed using GraphPad Prism (v.8.4.1, v.9.3.0) or R (v.4.2.3), RStudio software (v.1.3.1056). All values are reported as mean ± s.e.m. unless stated otherwise. Data were analysed using one-way or two-way ANOVA with Tukey’s, Dunnett’s or Šidák’s post-hoc tests where appropriate. Differences were considered to be significant when *P* < 0.05. Statistical significance of histological scores was evaluated using unpaired Mann–Whitney non-parametric tests. ANCOVA was used to correct for the influence of variability of covariates (for example, body mass) on main variates (for example, treatment). ANCOVA was performed and visualized using the HH package (v.3.1-47)^88^ in R and RStudio software after checking the homogeneity of regression slopes. The correlation analysis was performed using Pearson’s product-moment correlation.

### Reporting summary

Further information on research design is available in the Nature Portfolio Reporting Summary linked to this article.

## Online content

Any methods, additional references, Nature Portfolio reporting summaries, source data, extended data, supplementary information, acknowledgements, peer review information; details of author contributions and competing interests; and statements of data and code availability are available at 10.1038/s41586-023-06249-4.

## Supplementary information

Supplementary Information Supplementary Figs. 1–5.

Reporting Summary

## Data Availability

All data supporting the findings in this study are available within the Article and its Supplementary Information. The RNA-seq data of the liver tissue and tibialis anterior muscle have been deposited at the NCBI Gene Expression Omnibus (GEO) and are accessible under accession numbers GSE229708 (liver tissue) and GSE229794 (tibialis anterior muscle) or SuperSeries GSE230208. RNA-seq data of quadriceps samples from mice treated with β-2 agonist clenbuterol were downloaded from the NCBI Sequence Read Archive under reference number PRJNA756816. The data from GTEx Analysis V8 used for the analyses described in this paper were obtained from dbGaP accession number phs000424.v8.p2 on 11 May 2022 (https://www.gtexportal.org/home/). Gel source data are provided in Supplementary Fig. 4. Source data are provided with this paper.

## Source data

Source Data Fig. 1
Source Data Fig. 2
Source Data Fig. 3
Source Data Fig. 4
Source Data Extended Data Fig. 1
Source Data Extended Data Fig. 2
Source Data Extended Data Fig. 4
Source Data Extended Data Fig. 5
Source Data Extended Data Fig. 6
Source Data Extended Data Fig. 7
Source Data Extended Data Fig. 8
Source Data Extended Data Fig. 9
Source Data Extended Data Fig. 10
Source Data Extended Data Fig. 11

## Code availability

The code developed for RNA-seq analysis, bioinformatics analysis of the GTEx dataset and 2SMR are freely available at Zenodo (10.5281/zenodo.7838970).
