# Supplementary material for: GDF15 promotes weight loss by enhancing energy expenditure in muscle
Source: Nature. 2023 Jun 28;619(7968):143–50. doi: 10.1038/s41586-023-06249-4 (PMC10322716; doi:10.1038/s41586-023-06249-4)

nature portfolio

https://doi.org/10.1038/s41586-023-06249-4

Supplementary information

GDF15 promotes weight loss by enhancing energy expenditure in muscle

In the format provided by the authors and unedited

Supplementary Information Figure 1. Activity in CLAMS (related to Fig. 2A-E)

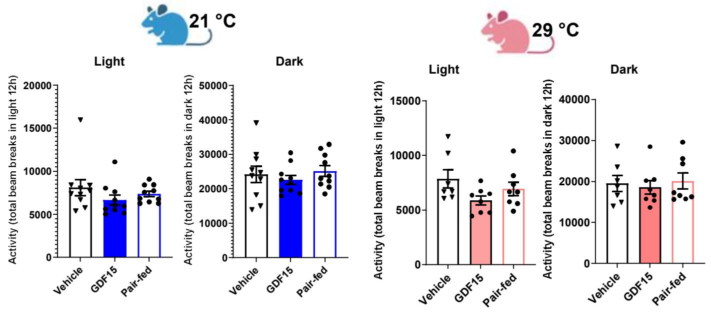

SI Figure 1. Animal activity during 12h-light/dark circle. Data are mean ± SEM, n = 10 mice/group. Mice illustrations are obtained from BioRender.com (publication license number TZ259QRW6B).

Supplementary Information Figure 2. A and B Gating strategy for flow cytometry analysis of immune cell populations stained with CD45, CD11b, F4/80, CD3 and CD4 antibodies. (related to Extended Data Fig. 5)

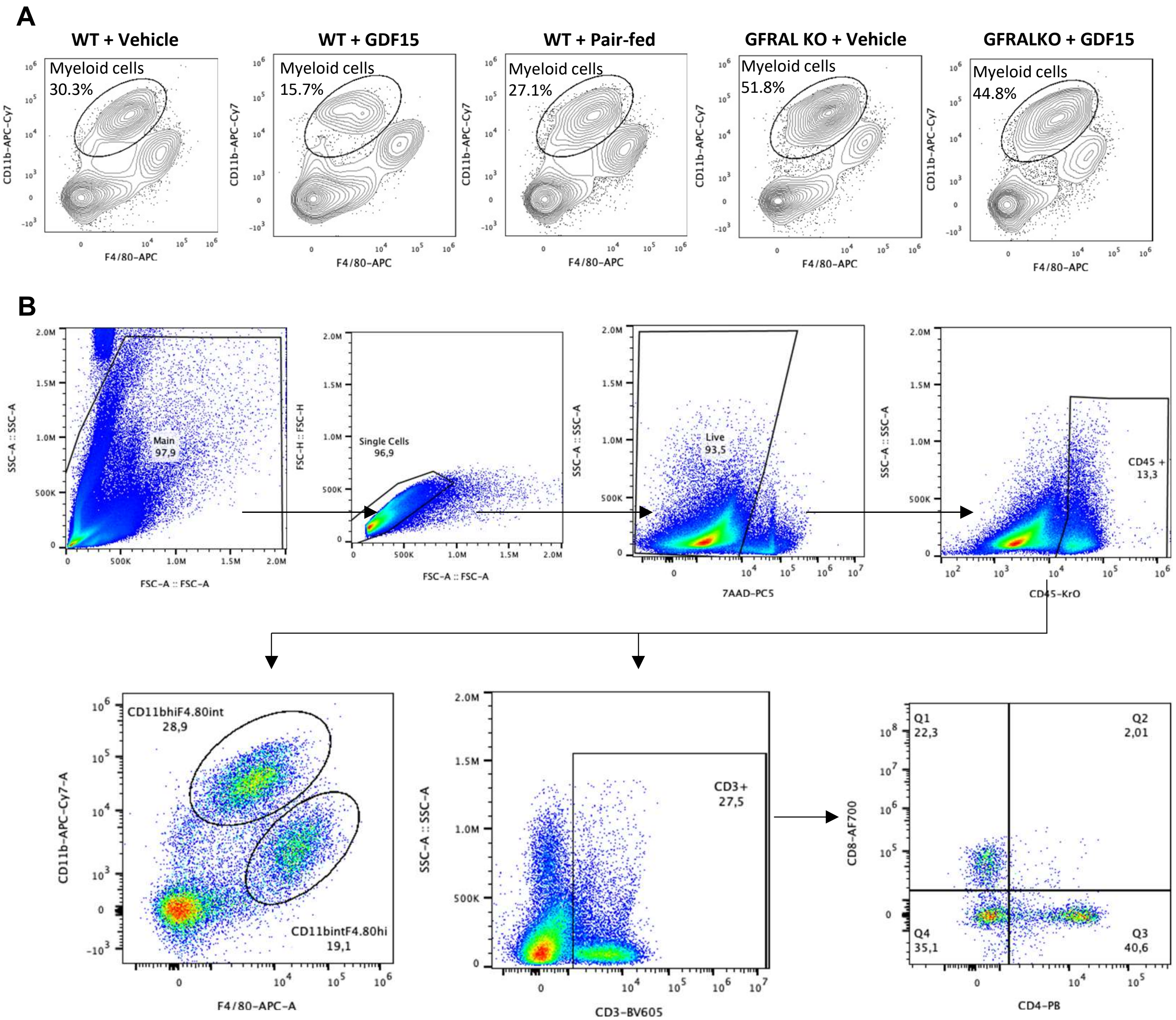

Supplementary Information Figure 3. Confirmation of denervation of BAT by injection with 6-hydroxydopamine hydrobromide (6OHDA) directly to BAT (related to Extended Data Fig. 9)

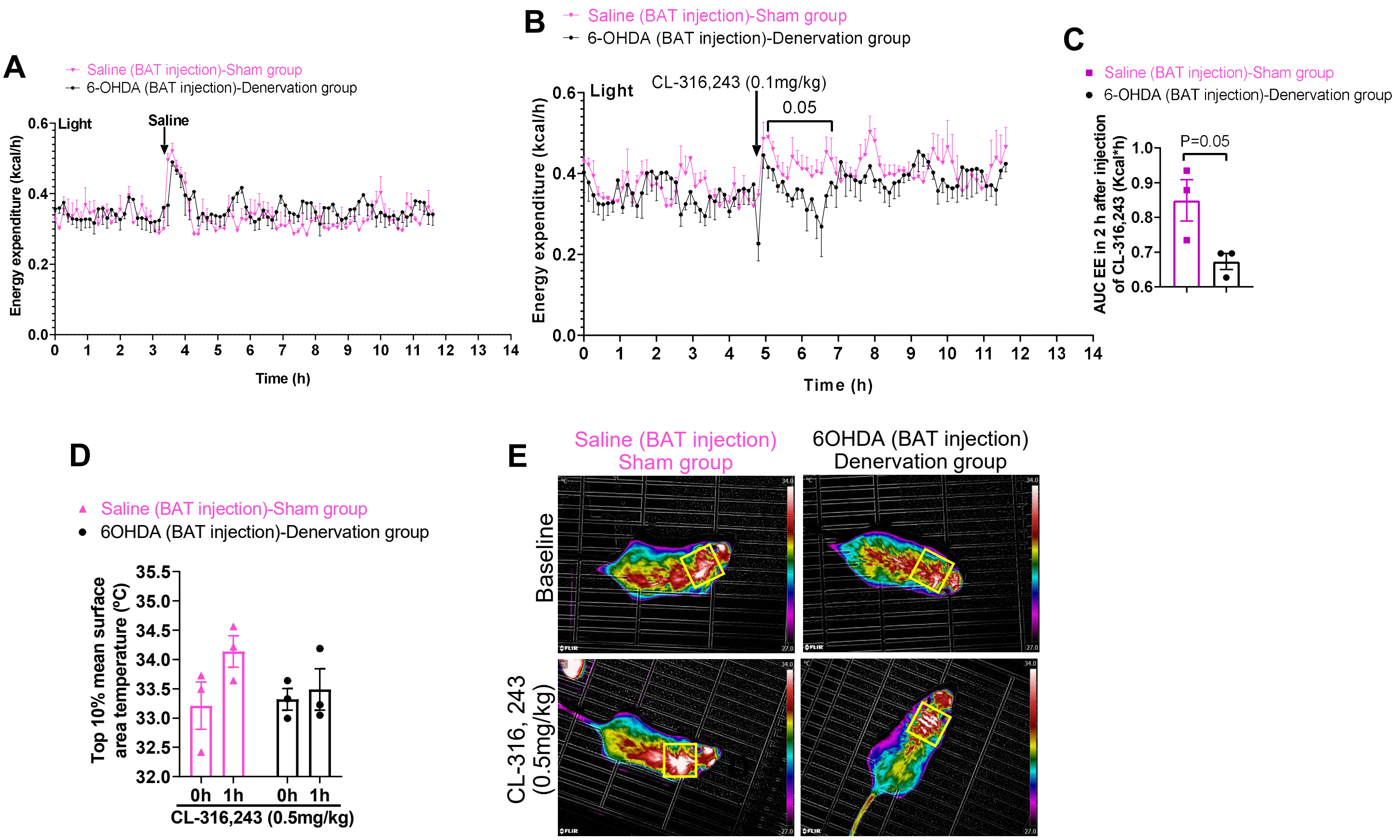

SI Figure 3. Confirmation of denervation of BAT by injection with 6-hydroxydopamine hydrobromide (6OHDA) directly to BAT. A and B, Experimental scheme for the effects of GDF15 and matched caloric restriction in pair-fed control on body weight, energy expenditure in C57BL/6J mice fed western diet and housed at thermoneutrality (TN, 29 °C). The BAT of mice were denervated by 6-hydroxydopamine hydrobromide (6OHDA). The BAT of mice was directly injected with 6-hydroxydopamine hydrobromide (6OHDA, BAT denervation group) or saline (Sham group). After 48 h of denervation, the mice were put in Comprehensive Laboratory Animal Monitoring System (CLAMS) and treated with saline on 1st day in CLAMS) (**A**) or beta-3 adrenergic agonist (CL-316,243, 0.1mg/kg, ip) on 2nd day in CLAMS (**B**). Compared to Sham group, BAT denervation abolished the effect of CL-316,243 on energy expenditure. Data are mean ± SEM, n = 3 mice/group. P values by multiple unpaired t test (two-sided). **C,** AUC of average energy expenditure in 2 h after injection of CL-316,243 (0.1mg/kg, ip). Data are mean ± SEM, n = 3 mice/group. P values by unpaired t test (two-sided). **D,** Top 10% mean surface area temperature in the interscapular BAT area of the mice. Data are mean ± SEM, n = 3 mice/group. **E,** Representative infrared images of mice. Images are displayed using the rainbow high contrast color palette in the FLiR Research IR program.

Supplementary Information Figure 4. Mitochondrial cleanliness checking and their related uncropped Immunblots (related to Extended Data Fig. 10H-K).

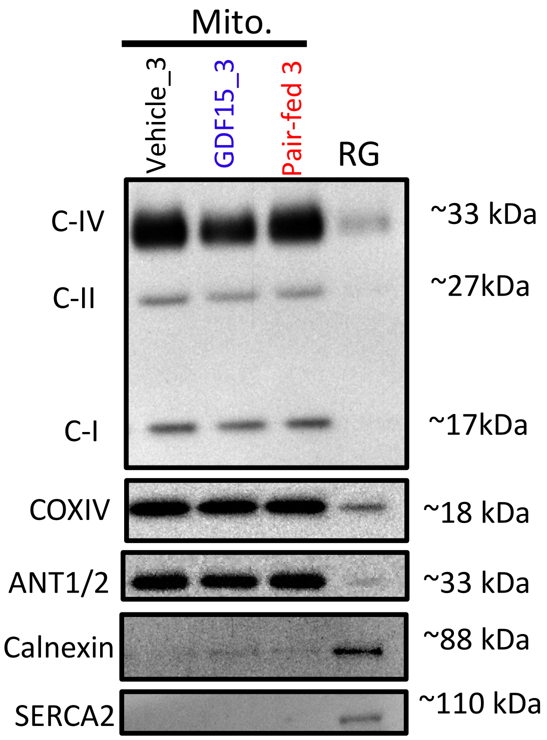

Uncropped Immunblots for C-IV, C-II, C-I

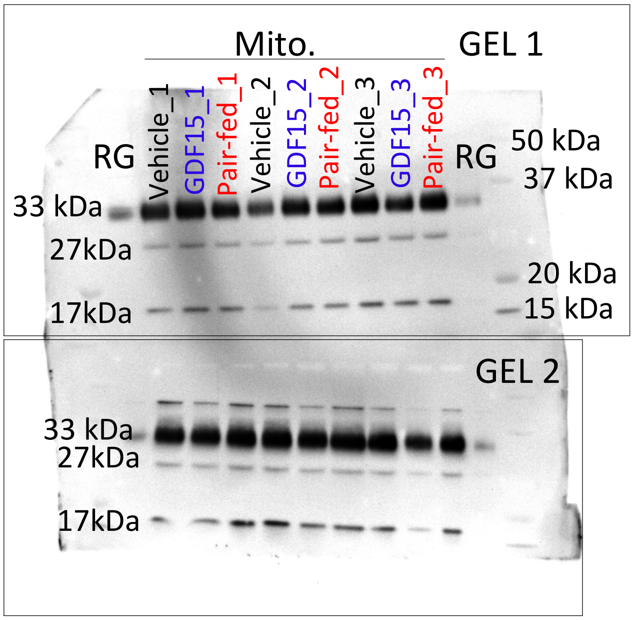

Vehicle_1: vehicle, mouse #1

GDF15_1: GDF15 treatment, mouse #1

Pair-fed_1: Pair-feeding group, mouse #1

Uncropped Immunblots for COXIV – Cytochrome C oxidase, subunit 4

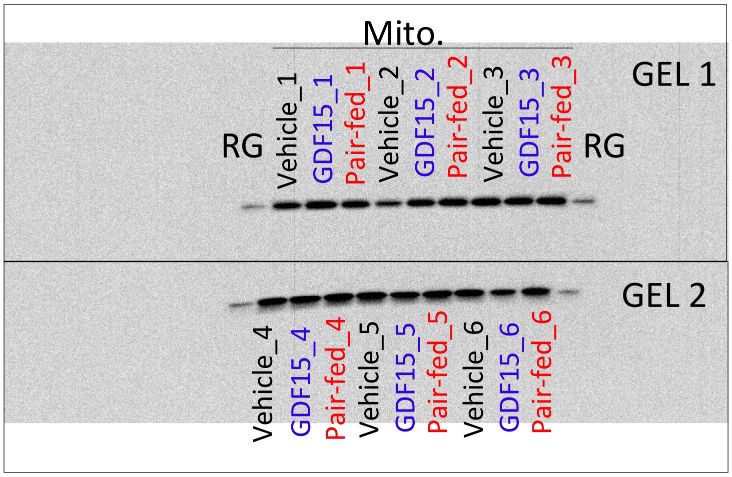

Uncropped Immunblots for ANT 1/2

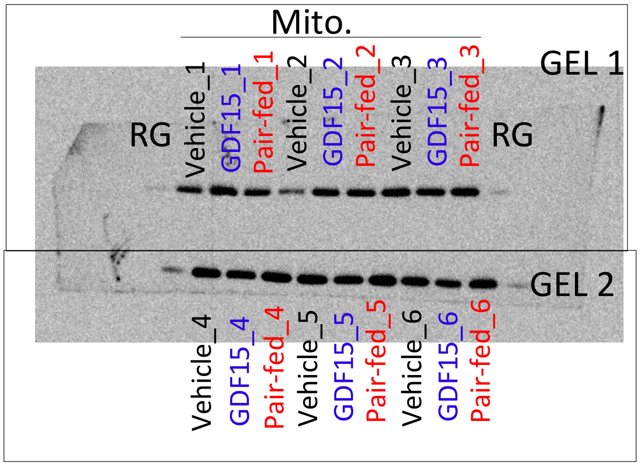

Uncropped Immunblots for Calnexin

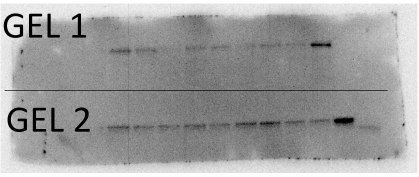

Uncropped Immunblots for SERCA2

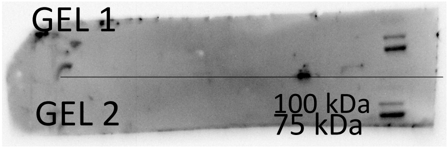

SI Figure 4. Mitochondrial cleanliness checking. mitochondrial proteins: COXI, COXIV; non-mitochondrial proteins: GLUT4, calnexin, SERCA2. (RG: red gastrocnemius muscle). Results were reproduced in 6 mice.

Supplementary Information Figure 5. Absolute JO2 in the permeabilized fibers during ADP titration. Data are means ± SEM, n = 6 mice/group except GDF15 group, n = 5 mice. We utilized the one-phase association curve from this ADP titration to estimate the ADP generated during the Ca2+ titration as an index of SERCA efficiency. (related to Figure 4G and Extended Data Fig. 10O).

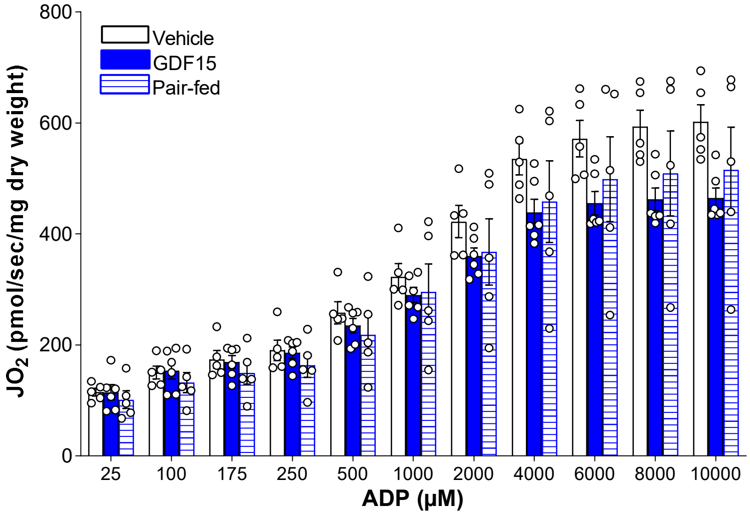

Supplement: Supplementary file 1 — Supplementary Figs. 1–5. [file 41586_2023_6249_MOESM1_ESM.pdf]
