# Supplementary material for: GDF15 promotes weight loss by enhancing energy expenditure in muscle
Source: Nature. 2023 Jun 28;619(7968):143–50. doi: 10.1038/s41586-023-06249-4 (PMC10322716; doi:10.1038/s41586-023-06249-4)
Supplement: Supplementary file 2 — Reporting Summary [file 41586_2023_6249_MOESM2_ESM.pdf]

nature portfolio

Corresponding author(s): Dr. Gregory Steinberg

Last updated by author(s): Apr 20, 2023

Reporting Summary

Nature Portfolio wishes to improve the reproducibility of the work that we publish. This form provides structure for consistency and transparency in reporting. For further information on Nature Portfolio policies, see our Editorial Policies and the Editorial Policy Checklist.

Statistics

For all statistical analyses, confirm that the following items are present in the figure legend, table legend, main text, or Methods section.

n/a	Confirmed	
☐	☒	The exact sample size (*n*) for each experimental group/condition, given as a discrete number and unit of measurement
☐	☒	A statement on whether measurements were taken from distinct samples or whether the same sample was measured repeatedly
☐	☒	The statistical test(s) used AND whether they are one- or two-sided<br>*Only common tests should be described solely by name; describe more complex techniques in the Methods section.*
☐	☒	A description of all covariates tested
☐	☒	A description of any assumptions or corrections, such as tests of normality and adjustment for multiple comparisons
☐	☒	A full description of the statistical parameters including central tendency (e.g. means) or other basic estimates (e.g. regression coefficient) AND variation (e.g. standard deviation) or associated estimates of uncertainty (e.g. confidence intervals)
☐	☒	For null hypothesis testing, the test statistic (e.g. *F*, *t*, *r*) with confidence intervals, effect sizes, degrees of freedom and *P* value noted<br>*Give P values as exact values whenever suitable.*
☒	☐	For Bayesian analysis, information on the choice of priors and Markov chain Monte Carlo settings
☒	☐	For hierarchical and complex designs, identification of the appropriate level for tests and full reporting of outcomes
☐	☒	Estimates of effect sizes (e.g. Cohen's *d*, Pearson's *r*), indicating how they were calculated

Our web collection on statistics for biologists contains articles on many of the points above.

Software and code

Policy information about availability of computer code

Data collection	Flowcytometry data were collected by CytoFLEX LX.<br>Dara from muscle function test were collected through via Dynamic Muscle Control and Analysis Software (version 615A, Aurora Scientific Inc.).<br>Immunofluorescence was visualized via Nikon Eclipse 90i microscope (Nikon; NY, USA).
Data analysis	Fro Two sample Mendelian Randomization analysis, we used TwoSampleMR R package (v0.5.6) from https://mrcieu.github.io/TwoSampleMR/.<br>For RNA-seq and transcriptomic analysis, we used MultiQC (v1.12), Trim Galore (0.6.5-1), Salmon (v1.10.1), DESeq2 (v1.38.3), GOstats (v 2.64.0), KEGG.db (v3.2.3), cnetplot packages for R.<br>FlowJo (Version 10.5) software was used to analyze data collected from Flow Cytometer.<br>Dara from muscle function test were analyzed via Dynamic Muscle Control and Analysis Software (version 615A, Aurora Scientific Inc.).<br>Immunofluorescence was analyzed using NIS-Elements AR software (Nikon, version 5.41.02).<br>Statistical analyses were performed using GraphPad Prism (version 8.4.1, version 9.3.0) or R (Version 4.2.3), RStudio software (Version 1.3.1056).

For manuscripts utilizing custom algorithms or software that are central to the research but not yet described in published literature, software must be made available to editors and reviewers. We strongly encourage code deposition in a community repository (e.g. GitHub). See the Nature Portfolio guidelines for submitting code & software for further information.

Data

Policy information about availability of data

All manuscripts must include a data availability statement. This statement should provide the following information, where applicable:

- Accession codes, unique identifiers, or web links for publicly available datasets
- A description of any restrictions on data availability
- For clinical datasets or third party data, please ensure that the statement adheres to our policy

All data supporting the findings in this study are available within the article, extended data and the supplementary information. The RNA-seq data of the liver tissue and tibialis anterior (TA) muscle have been deposited into the Gene Expression Omnibus (GEO) of the National Center for Biotechnology Information (NCBI) and are accessible through GEO Series accession number GSE229708 (for the liver tissue) and GSE229794 (the TA muscle). RNA-seq data of quadriceps samples from mice treated with beta-2 agonist clenbuterol were downloaded from the NCBI Sequence Read Archive under reference number RNA seq-PRJNA75681646 (https://www.ncbi.nlm.nih.gov/bioproject/PRJNA756816). The data from Genotype-Tissue Expression (GTEx) Analysis V8 used for the analyses described in this paper were obtained from: dbGaP accession number phs000424.v8.p2 on 11/05/2022 (https://www.gtexportal.org/home/). For gel source data, see Supplementary Information Figure 4. Source data are provided with this paper.
The developed code for RNAseq analysis, bioinformatics analysis of the Genotype-Tissue Expression (GTEx) dataset and 2SMR are freely accessed and obtained in Zonodo (https://zenodo.org/record/7838970#.ZD3uSXbMK39, DOI: 10.5281/zenodo.7838970).

Human research participants

Policy information about studies involving human research participants and Sex and Gender in Research.

Reporting on sex and gender	Not applicable
Population characteristics	Not applicable
Recruitment	Not applicable
Ethics oversight	Not applicable

Note that full information on the approval of the study protocol must also be provided in the manuscript.

Field-specific reporting

Please select the one below that is the best fit for your research. If you are not sure, read the appropriate sections before making your selection.

☒ Life sciences ☐ Behavioural & social sciences ☐ Ecological, evolutionary & environmental sciences

For a reference copy of the document with all sections, see nature.com/documents/nr-reporting-summary-flat.pdf

Life sciences study design

All studies must disclose on these points even when the disclosure is negative.

Sample size	Sample size for animal study were chosen following well-established protocols previously performed in our lab and others (Morrow MR et al., Cell Metab. 2022, PMID: 35675800; Desjardins EM et al., PNAS, 2022, PMID: 36409897; Day EA et al., Nat Metab., 2019, PMID: 32694673; Yang L et al, Nat Med. 2017, PMID: 28846099; Hsu JY et al., Nature. 2017, PMID: 28953886), and provide adequate power to detect the substantial effect, while also ensuring no more animals than necessary were used. In vitro sample sizes were based on previous experience (Rebalka IA et al., Am J Physiol Cell Physiol. 2019, PMID: 31509447). Sample sizes were also determined based on animal availability, homogeneity and consistency of characteristics in the selected models.
Data exclusions	One blood sample from 0.3 and 1 nmol/kg groups respectively at 8h in Figure 1B was lost due to technical error (bleeding problem). One sample point of Ucp3 and Sln gene expression in vehicle group in Figure 4D was lost due to technical error (The samples were not added in tubes accidentally, which caused no signal).
Replication	Chronic treatment of C57Bl6J mice with Vehicle, GDF15 and pairfeeding were performed at two different sites (McMaster University (ON, Canada) and Novo Nordisk (Maaloev, Denmark)).<br>Nearly all animal experiments were completed across 2 independent cohorts. All cell based studies are from 3 independent experiments.<br>All attempts at replication were successful.
Randomization	Prior to treatment, mice were randomized and separated into different treatment groups matched on body weight and composition.
Blinding	Investigators were blinded during tissue collection and histological and other analysis were blind for the investigators.

Reporting for specific materials, systems and methods

We require information from authors about some types of materials, experimental systems and methods used in many studies. Here, indicate whether each material, system or method listed is relevant to your study. If you are not sure if a list item applies to your research, read the appropriate section before selecting a response.

Materials & experimental systems

n/a	Involved in the study
☐	☒ Antibodies
☐	☒ Eukaryotic cell lines
☒	☐ Palaeontology and archaeology
☐	☒ Animals and other organisms
☐	☒ Clinical data
☒	☐ Dual use research of concern

Methods

n/a	Involved in the study
☒	☐ ChIP-seq
☐	☒ Flow cytometry
☒	☐ MRI-based neuroimaging

Antibodies

Antibodies used

Western blot: COXI (1:500, OXPHOS cocktail, Abcam Ab110413), COXIV (1:30000, Invitrogen A21347), GLUT4 (1:2500, Abcam Ab654), Calnexin (1:2000, Sigma C4731) and SERCA2 (1:1000, Abcam ab2861).
FACS antibody: Fc block (1:200, BD Biosciences, Catalog#: 553142), CD45.2 BV510 (1:25, BioLegend, Catalog#: 109838), CD11b APC-Cy7 (1:100, Invitrogen, Catalog#: A15390), F4/80-APC (1:100, Invitrogen, Catalog#: 17-4801-82), , CD3 BV605 (1:50, BD Biosciences, Catalog#: 563004), , CD4 PerCP-Cy5.5 (1:100, BD Biosciences, Catalog#: 550954), 7AAD (1:100, Thermofisher, Catalog#: A1310).

Validation

Antibodies for mouse GSDMB, mouse COXI, COXIV, GLUT4, Calnexin and SERCA2 used to detect proteins in Western Blot, have been validated by the corresponding manufacturers and the detailed information can be found on the manufacturers' websites.
Antibodies for mouse CD45.2 BV510, CD11b APC-Cy7, F4/80-APC, CD3 BV605, CD4 PerCP-Cy5.5, 7AAD for flow cytometer, have been validated by the corresponding manufacturers and the detailed information can be found on the manufacturers' websites.

Eukaryotic cell lines

Policy information about cell lines and Sex and Gender in Research

Cell line source(s)

C2C12 cell line was obtained from the American Type Culture Collection (ATCC, CRL-1772™).

Authentication

C2C12 cell line was authenticated by short tandem repeat (STR) profiling at ATCC.

Mycoplasma contamination

C2C12 cell line tested negative for mycoplasma contamination.

Commonly misidentified lines
(See ICLAC register)

No misidentified cell lines were used.

Animals and other research organisms

Policy information about studies involving animals; ARRIVE guidelines recommended for reporting animal research, and Sex and Gender in Research

Laboratory animals

Male C57Bl6J ordered from JAX, GDF15 KO and WT controls, GFRAL KO and WT controls and ADRB1,2,3 KO and WT controls treated with vehicle, GDF15 or pairfed as described in detail in manuscript. Mice were aged 8-42 weeks of age as described in methods. Mice were were placed on a NASH diet and housing at thermoneutral condition (~29°C) or ambient temperature (~21°C, 40-60%RH). at 8-weeks of age.

Wild animals

The studies did not include wild animals.

Reporting on sex

We only used male mice in our studies. Sex was not considered in study design.

Field-collected samples

The studies did not include field-collected animals.

Ethics oversight

All animals used in the study were housed and cared for in accordance with the local guidelines for Animal Use, and studies were approved by the Animal Ethics Research Board of McMaster University (AUP: 210104), Université de Sherbrooke (2021-3001), and Danish Animal Experiments Inspectorate (2020-15-0201-00756:C01).

Note that full information on the approval of the study protocol must also be provided in the manuscript.

Clinical data

Policy information about clinical studies

All manuscripts should comply with the ICMJE guidelines for publication of clinical research and a completed CONSORT checklist must be included with all submissions.

Clinical trial registration

The ClinicalTrials.gov identifier is NCT03221322.

Study protocol

Trial protocol for measuring Resting metabolic rate outlined in the published paper (Higher than predicted resting energy expenditure and lower physical activity in healthy underweight Chinese adults. Cell Metab. 2022 Oct 4;34(10):1413-1415. PMID: 35839758) and supplied on submission.
Trial protocol for measuring GDF15 and thyroid stimulating hormone (TSH) in human outlined in the published paper (Distinct skeletal muscle fiber characteristics and gene expression in diet-sensitive versus diet-resistant obesity. J Lipid Res . 2010 Aug;51(8):2394-404. PMID: 20332421).

Data collection

Resting metabolic rate (RMR) of 154 subjects was measured using a ventilated hood47 (JAEGER Oxycon Pro, Viasys Healthcare GmbH, Germany). The measurement was performed after an overnight fast between 8-10am. The hood was placed over the head of recumbent subjects. The measurement lasted for 40 min, during when the subjects were required to keep still yet remain awake. The mean values of every 10 minutes were then calculated and the minimum values were used as the RMR of the subjects.
For thyroid stimulating hormone (TSH) test, blood samples were collected after an overnight fast from women with obesity. TSH measurements were conducted by the Ottawa Hospital Laboratory Services (Ottawa, Ontario, Canada).

Outcomes

Resting metabolic rate (RMR) was adjusted with body composition from TANITA by using the published equation Ln BEE = -0.954 +0.707 Ln FFM +0.019 Ln FM48.

Flow Cytometry

Plots

Confirm that:

☒ The axis labels state the marker and fluorochrome used (e.g. CD4-FITC).

☒ The axis scales are clearly visible. Include numbers along axes only for bottom left plot of group (a 'group' is an analysis of identical markers).

☒ All plots are contour plots with outliers or pseudocolor plots.

☒ A numerical value for number of cells or percentage (with statistics) is provided.

Methodology

Sample preparation

For the preparation of liver cells, a lobe of the liver was collected following perfusion of the liver with PBS and digested with enzyme solution buffer containing 0.5 mg/ml pronase E, 0.088 U/ml collagenase D and 1% (vol/vol) DNase I for 30 min at 37°C. Single-cell suspension of liver non-parenchymal cells was prepared as previously described53, with a minor modification. Briefly, following digestion, the cells were filtered through a 100 μM cell strainer. After two centrifugation steps of 1 min at 50 g to remove hepatocytes, the remaining cells in suspension were further filtered through a 40 μM cell strainer. The non-parenchymal single cells were centrifuged at 1500 rpm for 5 min at 4°C before proceeding to blocking/antibody staining for flow cytometry. For isolation of SVCs, epididymal WAT was collected and minced into fine pieces and SVCs were isolated as previously described54. Briefly, minced samples were placed in RMPI culture media containing 1% BSA and an LPS-depleted collagenase cocktail (5401020001, Liberase TL Research Grade, Sigma) at a concentration of 0.03 mg/ml and were incubated at 37°C for 45 min. Once digestion was complete, samples were passed through a 100 μM cell strainer. The resulting cell suspension was centrifuged at 500g for 5 mins before proceeding to blocking/antibody staining for flow cytometry. RBCs were lysed using RBC lysis buffer (420301, Biolegend, USA) before proceeding to antibody staining for flow cytometry. For flow cytometry analysis, the cells were blocked with an antibody against Fc receptors and stained for 30 min on ice with an antibody cocktail as indicated in Methods part of manuscript.

Instrument

CytoFlex Flow Cytometer (Beckman Coulter Life Sciences, IN, USA)

Software

Flow cytometry data were collected via CytoFLEX LX. Data were analyzed by FlowJo (Version 10).

Cell population abundance

No cell sorting was performed.

Gating strategy

Based on the pattern of SSC-A/FSC-A, cells were gated excluding counting beads. Singlets were gated according to the pattern of FSC-H/ FSC-A. Dead cells were excluded using 7AAD staining. Positive populations were determined by the specific antibodies which were distinct from negative populations.

☒ Tick this box to confirm that a figure exemplifying the gating strategy is provided in the Supplementary Information.
